# A sensitive interactome-based approach to characterize drug activity in living human cells

**DOI:** 10.1016/j.jbc.2026.113557

**Published:** 2026-09-17

**Authors:** Cindy Kundlacz, Christelle Forcet, Nawal Hajj Sleiman, Samantha Vonau, Frédéric Marmigère, Benjamin Gillet, Sandrine Hughes, Laurent Gilquin, Hélène Duplus, Agnès Dumont, Gaël Yvert, Françoise Bleicher, Arnaud Gautier, Isabelle Coste, Toufic Renno, Samir Merabet

**Affiliations:** 1 Institut de Génomique Fonctionnelle de Lyon (IGFL), UMR5242, École Normale Supérieure de Lyon (ENSL), CNRS, Université Lyon-I, Lyon, France; 2 Institut Pasteur, Université Paris-Cité, CNRS-UMR 3528, Ingénierie des Anticorps, 25 Rue du Dr Roux, 75015 Paris, France. Institut Pasteur, Université Paris-Cité, UMR / INSERM - U1347, Unité de Parasitologie Moléculaire et Signalisation, Paris, France; 3 Laboratoire de Biologie et Modélisation de la Cellule (LBMC), UMR5239, École Normale Supérieure de Lyon (ENSL), CNRS, Université de Lyon, Lyon, France; 4 Sorbonne Université, Ecole Normale Supérieure, Université PSL, CNRS, Laboratoire des Biomolécules, LBM, Paris, France; 5 Université Claude Bernard Lyon1, INSERM U1052-CNRS UMR5286, Lyon Cancer Research Center, Centre Léon Bérard, Lyon, France

**Keywords:** MAPK, protein–protein interaction, cell-PCA, BiFC, splitFAST2, U0126, EI52

## Abstract

Abnormal protein–protein interactions (PPIs) involving signaling proteins contribute to numerous human diseases. Targeting such aberrant PPIs with selective inhibitory molecules is a promising therapeutic strategy, yet assessing how many PPIs are affected by a candidate drug, and whether inhibition occurs before or after complex assembly, remains technically challenging. Here, we present a live-cell methodology that captures drug-sensitive PPIs by integrating drug-induced cellular responses with interaction mapping. As a proof of concept, we identify extracellular signal–regulated kinase 1 PPIs that are selectively altered by a novel chemical molecule exhibiting antitumor efficacy. We further apply complementary real-time imaging tools to visualize the dissociation of preassembled extracellular signal–regulated kinase 1–cofactor complexes in living cells. Our approach provides a generalizable framework for decoding drug activity on PPIs for virtually any protein target, enabling more precise and physiologically relevant evaluation of therapeutic mechanisms.

Cell–cell communication plays a pivotal role in determining cell fate and function and relies on diverse signaling molecules that bind to specific receptors in the plasma membrane. This receptor–ligand interaction initiates intracellular phosphorylation cascades, culminating in the activation of specific gene responses within the nucleus. One of the most widespread and conserved core phosphorylation cascades is the Ras (rat sarcoma virus protein family)–RAF (rapidly accelerated fibrosarcoma protein kinase family)–mitogen-activated protein kinase kinase (MEK)–extracellular signal–regulated kinase (ERK) (mitogen-activated protein kinase [MAPK]) signaling pathway, which is activated by receptor tyrosine kinase receptors in response to various extracellular stimuli (*e*.*g*., growth factors; Fig. 1*A*) (1). The Ras–RAF–MEK–ERK axis is involved in the regulation of fundamental cellular processes, including proliferation, growth, differentiation, migration, and apoptosis. The central role of the MAPK signaling pathway is illustrated by the number of cancers that result from its dysregulation in human (2). Consequently, extensive research efforts have focused on identifying small-molecule inhibitors targeting the Ras–RAF–MEK–ERK axis to counteract its aberrant activation in cancer (3, 4). The majority of these inhibitors primarily target RAF, but their therapeutic application is limited by the observation of MAPK signaling reactivation and cancer resistance post-treatment (5, 6). Fewer inhibitory molecules target the MEK family members, MEK1 and MEK2 (thereafter denoted as MEK), or ERK1 and ERK2 (thereafter denoted as ERKs). Still, there is growing consensus that combining RAF and MEK–ERK inhibitors could constitute a promising therapeutic strategy against the recurrence of MAPK-driven tumors (5, 6).

**Figure 1:**
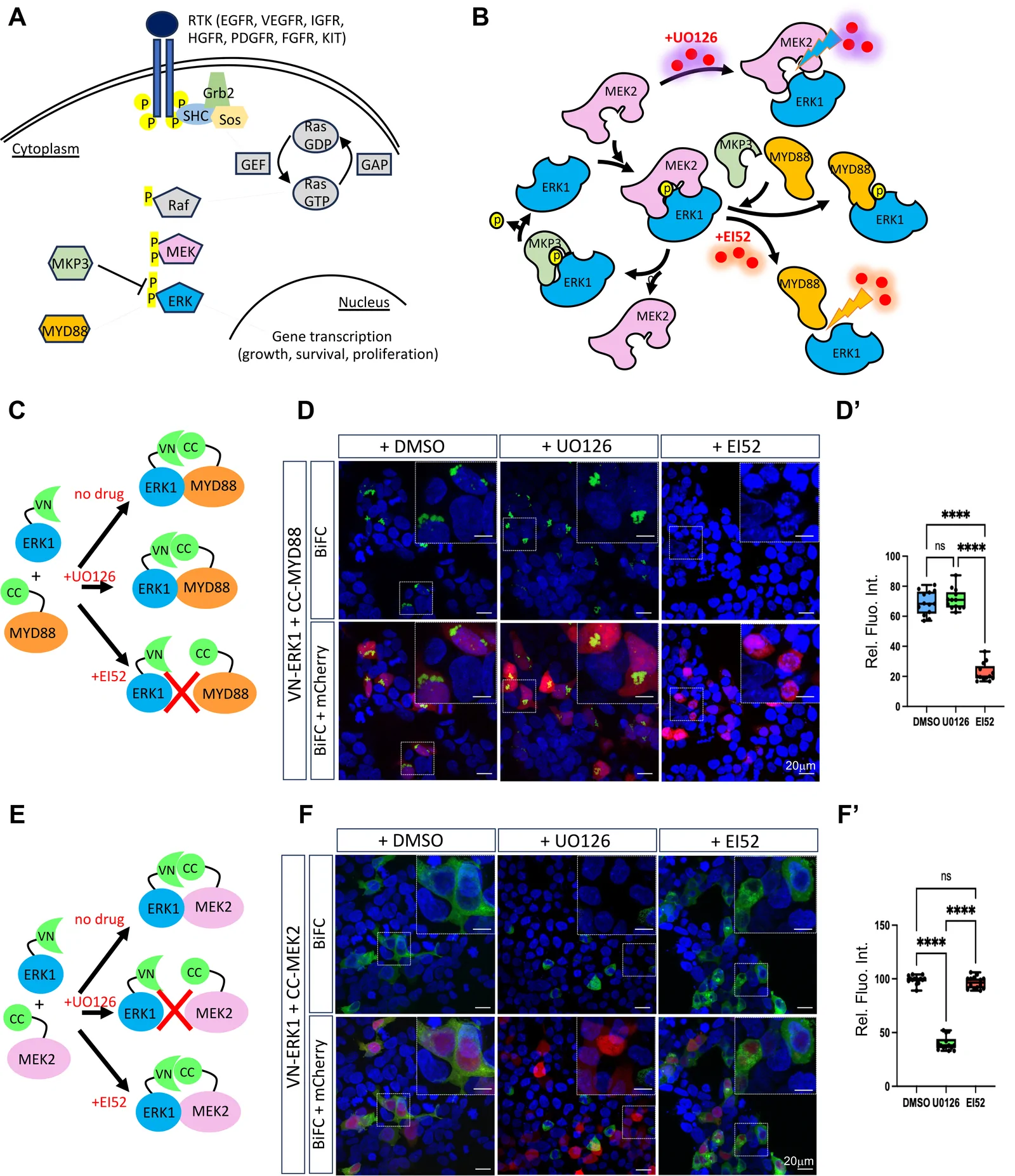
**BiFC validation of the specific effect of U0126 and EI52 inhibitory molecules on their respective ERK1–cofactor complexes in live HEK293T cells.***A*, different ligands can activate the ERK pathway through binding to a receptor tyrosine kinase. The phosphorylation of ERK by its upstream regulator MEK is inhibited by MKP3 and, reversely, protected by binding to MyD88 (11). *B*, role of the U0126 and EI-52 inhibitory molecules in the context of ERK1–MEK2 or ERK1–MyD88 complex, respectively. *C* and *D*, validation of the effect of EI-52 on ERK1–MyD88 interaction, as assessed by BiFC in live HEK293T cells. *C*, scheme of the different conditions. The test with U0126 serves as a negative control. *D*, illustrative confocal acquisitions of BiFC signals resulting from the interaction between VN–ERK1 and CC–MyD88 in live HEK293T cells in the presence of DMSO, DMSO and U0126, or DMSO and EI-52, as indicated. *E* and *F*, specificity control of EI52 on ERK1–MEK2 interaction, as assessed by BiFC in live HEK293T cells. *E*, scheme of the different conditions. *F*, illustrative confocal acquisitions of BiFC signals resulting from the interaction between VN–ERK1 and CC–MEK2 in live HEK293T cells in the presence of DMSO, DMSO and U0126, or DMSO and EI-52, as indicated. Graphs on the *right* represent the quantification of the BiFC signal (relative to mCherry as a transfection efficiency reporter) in the different conditions. Significance was determined relative to the mean BiFC value calculated with the control DMSO condition and was evaluated using *t* test (two conditions) or one-way ANOVA (more than two conditions). Minimum number of biological replicates n = 3; ∗∗∗∗*p <* 00.001; ns, nonsignificant. Scale bars represent 20 μm and 10 μm (in the enlargements). See also Fig. S1 and the Experimental procedures section. BiFC, bimolecular fluorescence complementation; CC, C-terminal fragment of cyan fluorescent protein; DMSO, dimethyl sulfoxide; ERK1, extracellular signal–regulated kinase 1; HEK293T, human embryonic kidney 293T cell line; MEK, mitogen-activated protein kinase kinase; VN, N-terminal fragment of Venus.

One of the first described MEK inhibitors is a chemical compound called U0126. It was identified from a screen for molecules that could suppress the expression of an AP-1-driven luciferase reporter in COS7 cells (7). U0126 blocks the kinase activity of MEK on ERK (Fig. 1*B*). Its allosteric mode of action was deduced from similar small inhibitors (like CI-1040/PD184352), with binding to a unique hydrophobic pocket adjacent to but not overlapping with the ATP- and ERK-binding sites of MEK. X-ray structures further showed that the mechanism of action of PD184352-like inhibitors was to bind and stabilize a naturally inactive conformation of MEK2. In particular, the N-terminal region of the MEK2 activation loop was under inactive alpha-helical conformation that partially occluded the binding site for the ERK activation loop (8, 9). U0126 has been reported to reduce cell proliferation in various cancer contexts and to also affect apoptosis, autophagy, and inflammation in other cell contexts (10).

Another partner of ERKs, called MyD88, has been described to compete for binding with the phosphatase MKP3, allowing the maintenance of the phosphorylated state of active ERKs (11). MyD88 was found to be overexpressed and to interact *via* its D domain with the D-recruitment site of ERKs in several cancer contexts, as well as to protect cancer cells against genotoxic agents (11). Therefore, MyD88 represents a novel player in MAPK signaling for cell cycle control and cell transformation, constituting a promising target of choice for therapeutic development in MAPK-dependent cancers. Accordingly, an inhibitory molecule called EI-52 has been developed to inhibit the interaction between the ERK1 isoform and MyD88 (Fig. 1*B*). This molecule has recently been shown to trigger immunogenic apoptotic cancer cell death *in vitro* and elicit an antitumor T-cell response *in vivo* (12).

Beyond targeting the ERK1–MyD88 complex, the complete interactome-wide specificity and mechanism of action of EI-52 require further investigation. As a proof of concept, we proposed a novel experimental approach to identify more broadly ERK1 interactions that could be sensitive to EI-52, using U0126 as a specificity control. To this end, we generated a library of 900 different candidate human ORFs (hORFs). We called this library “BiFC–EI-52–ERKeome” as it is compatible for bimolecular fluorescence complementation (BiFC) and contains genes involved in the regulation of the MAPK signaling pathway and genes of cellular functions induced by EI-52. This work led to the identification of novel ERK1 interactions that are specifically inhibited by EI-52. Moreover, to better characterize the inhibitory potential of EI-52, we developed a complementary innovative method that allows visualizing its capacity to disrupt the target protein–protein interactions (PPIs) after their formation in living cells.

## Results

### Validation of the cell system

Our experimental strategy is based on the BiFC technology, which allows visualizing PPIs in living cells. BiFC relies on the property of monomeric fluorescent proteins to be reconstituted from two separate hemifragments upon spatial proximity (13). Given that our goal was to establish an ERKeome for performing a drug-sensitive screen, we first confirmed the suitability of BiFC in this perspective. More specifically, we tested whether BiFC could recapitulate the EI-52-sensitive interaction of ERK1 with MyD88, as compared with the ERK1–MEK2 interaction. As specificity controls, we also tested the effect of U0126 on these interactions. Practically, ERK1 was fused to the N-terminal fragment of the GFP Venus (VN), whereas MyD88 and MEK2 were fused to the C-terminal fragment of the blue fluorescent protein Cerulean(CC, Fig. 1, *C* and *E*). The VN and CC fragments can complement each other, producing a VN-like fluorescent signal (14). All constructs were placed under the same doxycycline (dox)-inducible promoter (see the Experimental procedures section), and a red mCherry reporter vector was added to normalize BiFC with transfection efficiency between the different conditions. Transfection of each construct in human embryonic kidney 293T (HEK293T) cells showed clear distinct subcellular locations. VN–ERK1 was homogeneously located in the cytoplasm and nucleus, underlining that it could exist as both inactive (nonphosphorylated in the cytoplasm) and active (phosphorylated in the nucleus) forms in HEK293T cells (Fig. S1). In contrast, CC–MyD88 concentrated in a few cytoplasmic foci juxtaposed to the nucleus, whereas CC–MEK2 was exclusively located in the cytoplasm (Fig. S1). These profiles were in accordance with previously described subcellular localizations or ERK1, MyD88, and MEK2 in other cell types (12). Incubation with EI-52 (at the concentration used in previous work (12), see also the Experimental procedures section) did not change the subcellular localization of VN–ERK1 when compared with the no-drug condition (Fig. S1). Interestingly, treating the cells with U0126 (at a concentration determined from prior work (7), see also the Experimental procedures section) affected the localization of VN-ERK, which was less enriched in the nucleus than in the absence of the drug (Fig. S1). This observation suggests that U0126 affects more strongly the phosphorylation of ERK2 than EI-52.

Transfection of VN–ERK1 with either CC–MyD88 or CC–MEK2 resulted in a BiFC fluorescent signal that reproduced the subcellular localization of MyD88 or MEK2, showing that the two cofactors were able to concentrate ERK1 at their respective specific subcellular locations (Fig. 1, *D* and *F*). Importantly, incubating the cells with EI-52 led to a strong decrease in the ERK1–MyD88 BiFC signals (80% loss on average), whereas no effect was observed when cells were in the presence of U0126 (Fig. 1, *D* and *D′*). In contrast, EI-52 had no effect on ERK1–MEK2 BiFC signals, whereas U0126 had clear inhibitory effects (60% loss on average, Fig. 1, *F* and *F′*). This result confirms the destabilizing effect of U0126 deduced from X-ray structures of ERK–MEK complexes bound with similar inhibitors (8).

Altogether, these preliminary experiments validated that our experimental BiFC conditions were appropriate to recapitulate the specific effect of EI-52 and U0126 on ERK1 interaction properties.

### Modifying experimental parameters of the original cell-protein complementation assay–based strategy to gain specificity and reproducibility

The Cell-PCA (protein complementation assay) strategy consisted in using a cell line expressing around 5000 hORFs fused to the CC fragment for performing successive BiFC screens and comparing interactomes with different VN-fusion bait proteins in the same cellular context (15). In the present work, we considerably modified some experimental parameters of Cell-PCA to make the approach more accessible, specific, and reproducible.

The first parameter was the establishment of a biologically relevant CC-ORFeome, which contrasted with the previous large-scale design of Cell-PCA. Here, it consisted in generating a library of CC-ORFs covering functions related to cellular pathways that are regulated by ERK1 and modified upon treatment with EI-52 (12, 16). More specifically, we selected 984 genes from the human ORFeome v8.1 that were associated with MAPK signaling and functions induced by EI-52, such as apoptosis, immune pathways, and inflammatory response (Fig. 2*A*). Several of these functions are also induced by U0126, making the BiFC–EI-52–ERKeome library also appropriate for screening U0126-sensitive interactions to some extent. It should be noted that several MAPK pathway components (such as MEK2) are absent from the human ORFeome v8.1, precluding their transfer to the BiFC–EI-52–ERKeome library. The 984 selected ORFs were cloned in fusion with the CC fragment and used to establish an HEK293T cell population carrying the BiFC–EI-52–ERKeome library. This cell population eventually contained 907 integrated CC-ORFs after sequencing (Fig. 2*A* and Table S1, see also the Experimental procedures section), of which 95 were described as ERK1 interactors in the BioGRID database (corresponding to 10.47% of the BiFC–EI-52–ERKeome library; https://thebiogrid.org/111581/summary/homo-sapiens/mapk3.html and Fig. S2).

**Figure 2:**
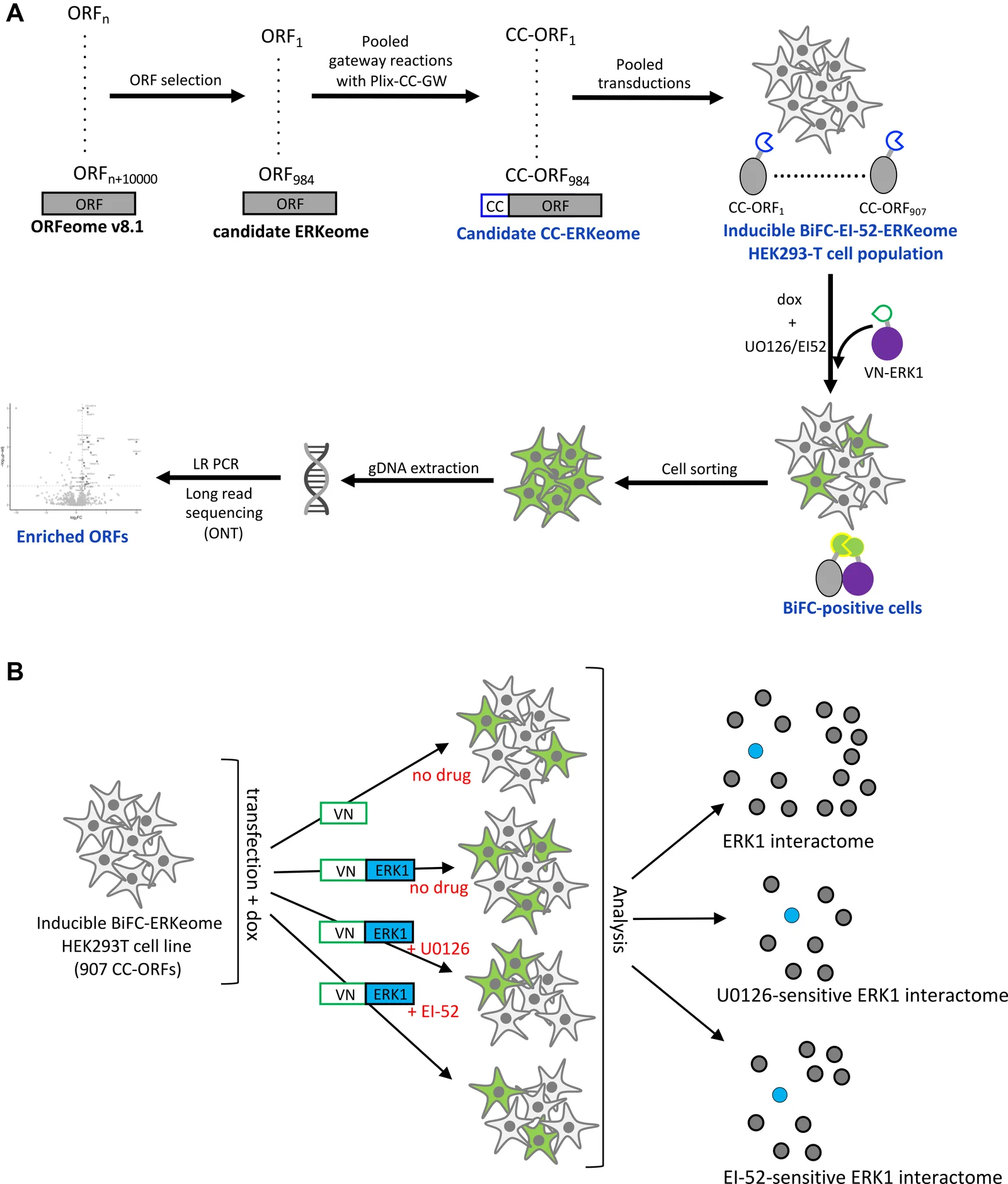
**Principle of the Cell-PCA strategy.***A*, establishing a novel BiFC–EI-52–ERKeome cell population to perform drug-sensitive BiFC screens with ERK1. Each step relies on new tools for improved specificity and robustness when compared with the original Cell-PCA approach (15). See main text for details. *B*, experimental conditions for identifying drug-sensitive interactions of VN–ERK1. See also Figs. S2 and S3 and Table S1. ERK1, extracellular signal–regulated kinase 1; PCA, protein complementation assay; VN, N-terminal fragment of VN.

The second parameter that we modified was the expression system. A major drawback of Cell-PCA was the leakiness of the integrated CC-ORFeome (that was under the control of the Tet-off system (15)), which prevented the maintenance of a stable cell line over several generations. Therefore, we built another vector where each CC-ORF is under the Tet-on promoter. This vector also expresses the most-recent generation of reverse tetracycline-controlled transactivator (rtTA) (rtTA-advanced) that is more sensitive to the dox and has lower background than the original rtTA (Fig. S3). Altogether, these tools make the BiFC–EI-52–ERKeome cell population highly stable over several generations, allowing successive screens with several biological replicates.

The third parameter that we implemented was the selection procedure. In the previous version of Cell-PCA, the selection of positive interacting ORFs was based on a frequency enrichment score when comparing the sorted fluorescent cells to the nonsorted cold cells. Here, BiFC screens were systematically performed with an additional condition corresponding to the VN fragment alone (Fig. 2*B*). This condition was considered as a reference threshold since the BiFC that could result from the association between VN and the CC-ORF gave a proxy for both the CC-ORF expression level and integration frequency in the BiFC cell population. Moreover, the comparison with VN alone allowed selecting interactions with a frequency score that was higher than the reference threshold, thus reflecting an increase in interaction affinity because of the specific interaction with the VN-bait fusion protein.

The last modified parameter was the identification of positive ORFs with a novel long-read sequencing protocol by Oxford Nanopore Technologies. It enabled obtaining the complete integrated CC-ORFs with fewer amplification cycles. The identification of positive CC-ORFs was achieved by establishing a novel analytical pipeline for automated application of statistics on read counts (see the Experimental procedures section).

### Identifying novel interactions of ERK1 with Cell-PCA in live HEK293T cells

Before starting the cell sorting, we first validated that the HEK293T cell population harboring the BiFC–EI-52–ERKeome library did not produce fluorescent cells either with dox or upon transfection of VN–ERK1 in the absence of dox (Fig. S4). In contrast, between 5% and 10% of cells became fluorescent upon transfection of the VN fragment or VN–ERK1 construct in the presence of dox, with or without the drug (Fig. S4 and the Experimental procedures section). A total of 50,000 fluorescent cells were sorted in each condition, and the different conditions (no drug *versus* in the presence of EI-52 or U0126) were applied to the same batch of cells in each biological replicate. A total of three biological replicates were performed (see the Experimental procedures section and raw data provided in Tables S2).

PCA analysis in the absence of drugs confirmed the correlation among the replicates and that the VN and VN–ERK1 conditions clustered separately (Fig. S5). We then applied a modified DESeq2 pipeline, which uses a model based on the negative binomial distribution allowing robust estimation of fold changes (FCs) and significant differential enrichment, to automatically identify ORFs significantly enriched in association with VN–ERK1 when compared with VN alone. The selection was based on the adjusted *p* value (equal to or less than 0.05 and calculated by considering all conditions: see the Experimental procedures section) and on the log2FC (arbitrarily set at a minimum of 0.70 to consider a significant enrichment when compared with the VN fragment). In total, 26 candidate cofactors met these selection criteria in the absence of drug (Fig. 3*A* and Tables S3 and S4). Half of these candidate cofactors form a physical and functional cluster related to MAPK signaling (Fig. 3*B*), which was also the most enriched Gene Ontology (GO) term (Fig. 3*C*). Thus, although the BiFC–EI-52–ERKeome library was more enriched in GO terms related to the effects induced by EI-52, the interactome of ERK1 reproduced expected GO terms for the canonical MAPK signaling in the absence of the drug. Interestingly, the majority of the positive candidates (18/26) are predicted to contain an ERK-binding domain, thus reinforcing their potential to interact with ERK1 (https://scansite4.mit.edu/#home and Fig. S6). Finally, 7 of the 26 positive candidates were already described as ERK1 interactors in databases (Fig. S6 and Table S4). The over-representation analysis (ORA, (17)) confirmed that this number of seven known interactors constitutes a significant enrichment when considering the BiFC–EI-52–ERKeome library and its total number of known ERK interactors (see the Experimental procedures section and Table S5).

**Figure 3:**
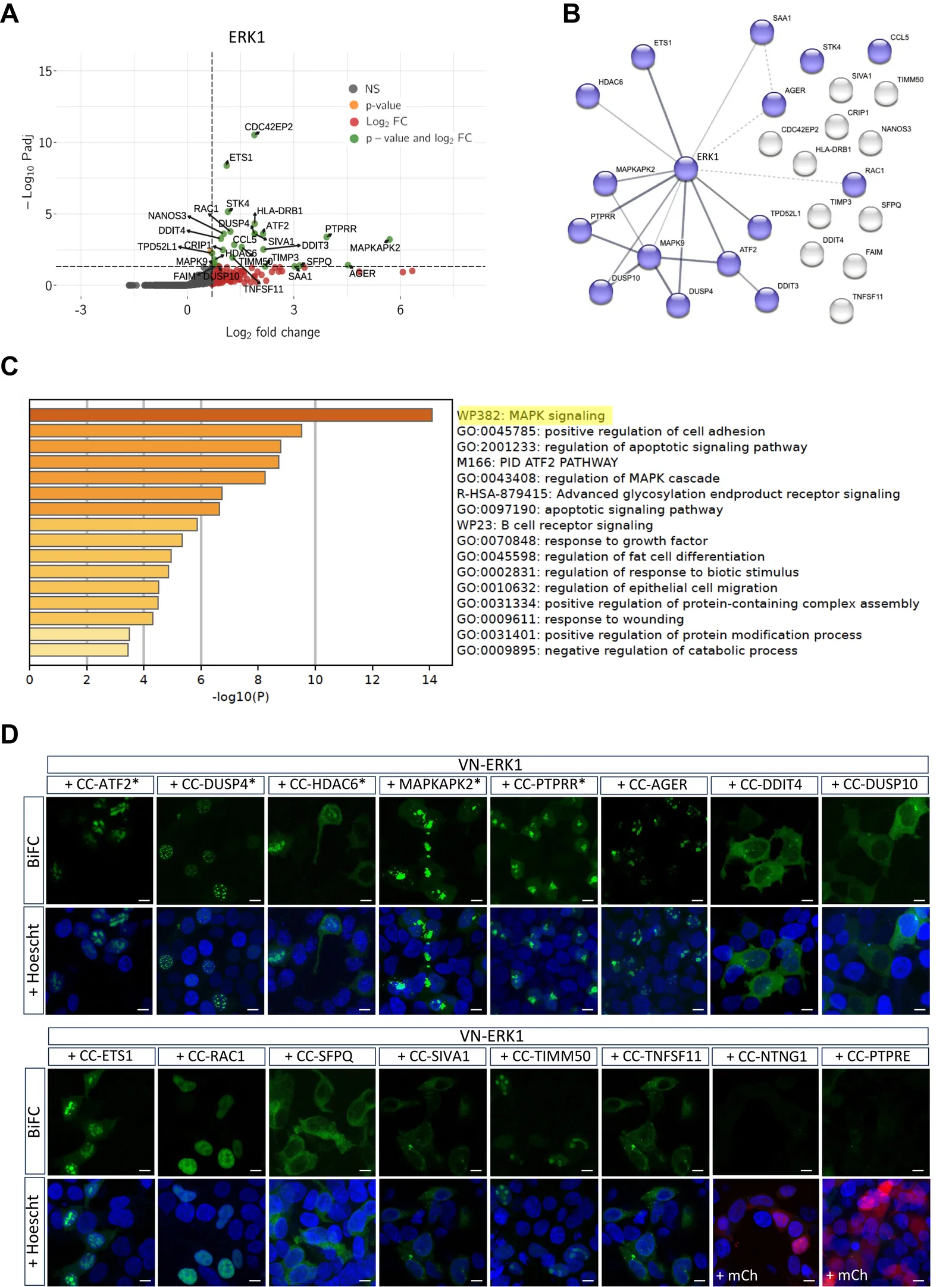
**Capture of the ERK1 interactome with the Cell-PCA strategy.***A*, Volcano plot of ORFs of the BiFC–EI-52 ERKeome distributed as a function of log2 FCs and adjusted *p* values when comparing the results between VN-ERK1 and VN. Selected interactions that met the log2FC (equal or superior to 0.70: dotted line on *x*-axis) and adjusted *p* value cutoff (in logarithmic scale, that is –Log10[0.05], dotted line *y*-axis) are highlighted in *green* and labeled. Interactions that met only the adjusted *p* value or Log2FC criterion are highlighted in *orange* or *red*, respectively. See also the Experimental procedures section. *B*, clustering of ERK1-positive interactions with STRING. Interactions involved in the MAPK signaling pathways are highlighted in *blue*. *Solid lines* are proportional to the confidence score (the minimum required interaction score was set at medium confidence) and denote functional and physical interactions with ERK1 (see also the Experimental procedures section). *C*, GO-term enrichments as deduced from the ERK1 interactome (see also the Experimental procedures section). *D*, individual BiFC assays confirming the ERK1-positive or ERK1-negative interaction status of selected ORFs from the Cell-PCA screen. VN–ERK1 was individually transfected with the candidate CC-ORF, and the interaction was analyzed in live HEK293T cells. Hoechst (*blue*) stains the nucleus. The mCherry transfection reporter is shown only for the two BiFC-negative ORFs (NTNG1 and PTPRE). *Stars* denote candidates already described as ERK1 cofactors in databases. Pictures are illustrative confocal acquisitions from at least two different experiments. The scale bar represents 10 μm. See also Experimental procedures section (Figs. S4–S6 and Tables S2–S5). BiFC, bimolecular fluorescence complementation; ERK1, extracellular signal–regulated kinase 1; FC, fold change; GO, Gene Ontology; HEK293T, human embryonic kidney 293T cell line; MAPK, mitogen-activated protein kinase.

To validate our list of potential ERK1-interacting proteins, we performed individual BiFC assays with 14 ERK1-positive (5 known ERK1 interactors and 9 novel interactions chosen to cover various functions) and 2 ERK-negative ORFs (randomly taken from the BiFC–ERKeome library) in living HEK293T cells (Fig. 3*D*). Interactions were all confirmed, and the analysis showed various and specific interaction profiles in the nucleus and/or cytoplasm, illustrating the diversity of contacts that could be engaged by the active or inactive form of ERK1 in HEK293T cells (Fig. 3*D*).

Overall, the screen with VN–ERK1 in the HEK293T cell population harboring the BiFC–EI-52–ERKeome revealed a limited but significant number of positive interactions (26 of 907), highlighting the specificity of the approach.

### Identification of novel ERK1 interactions sensitive to EI52 and U0126

In the absence of drug, the comparison between VN and VN–ERK1 led to the identification of 26 interactions that were significantly enriched with ERK1. The screen with VN–ERK1 was also performed in parallel by preincubating the BiFC–EI-52–ERKeome cell population with EI-52 or U0126 (Fig. 2*B* and the Experimental procedures section). The PCA analysis confirmed the distinct global effects of the two drugs on ERK1 interaction properties (Fig. S5). The same criterion of adjusted *p* value (≤0.05) and log2FC (set at a minimum of 0.7) was applied to select interactions that were impacted by the drug treatment when compared with the no-drug condition.

We first analyzed whether the two drugs could induce ectopic interactions with candidates previously identified as negative ERK1 interactors by Cell-PCA. To this end, we considered the full BiFC–EI-52–ERKeome. The analysis showed that EI-52 could induce 30 new interactions that overall correspond to EI-52-induced related GO terms (Tables S6 and S7 and Fig. S7). In contrast, U0126 did not induce any new interactions (Table S8 and Fig. S7).

Next, we considered the 26 ERK1-positive interactors to determine whether these interactions could be lost in the presence of the drug. The analysis showed that three interactions were significantly affected by either EI-52 (Table S9) or U0126 (Table S10). Interestingly, each set of affected interactions was different for the two drugs (Fig. 4, *A*–*C*). We also noticed that none of the 26 ERK1 interactions was found to be more enriched in the presence of EI-52 or U0126 (Tables S9 and S10).

**Figure 4:**
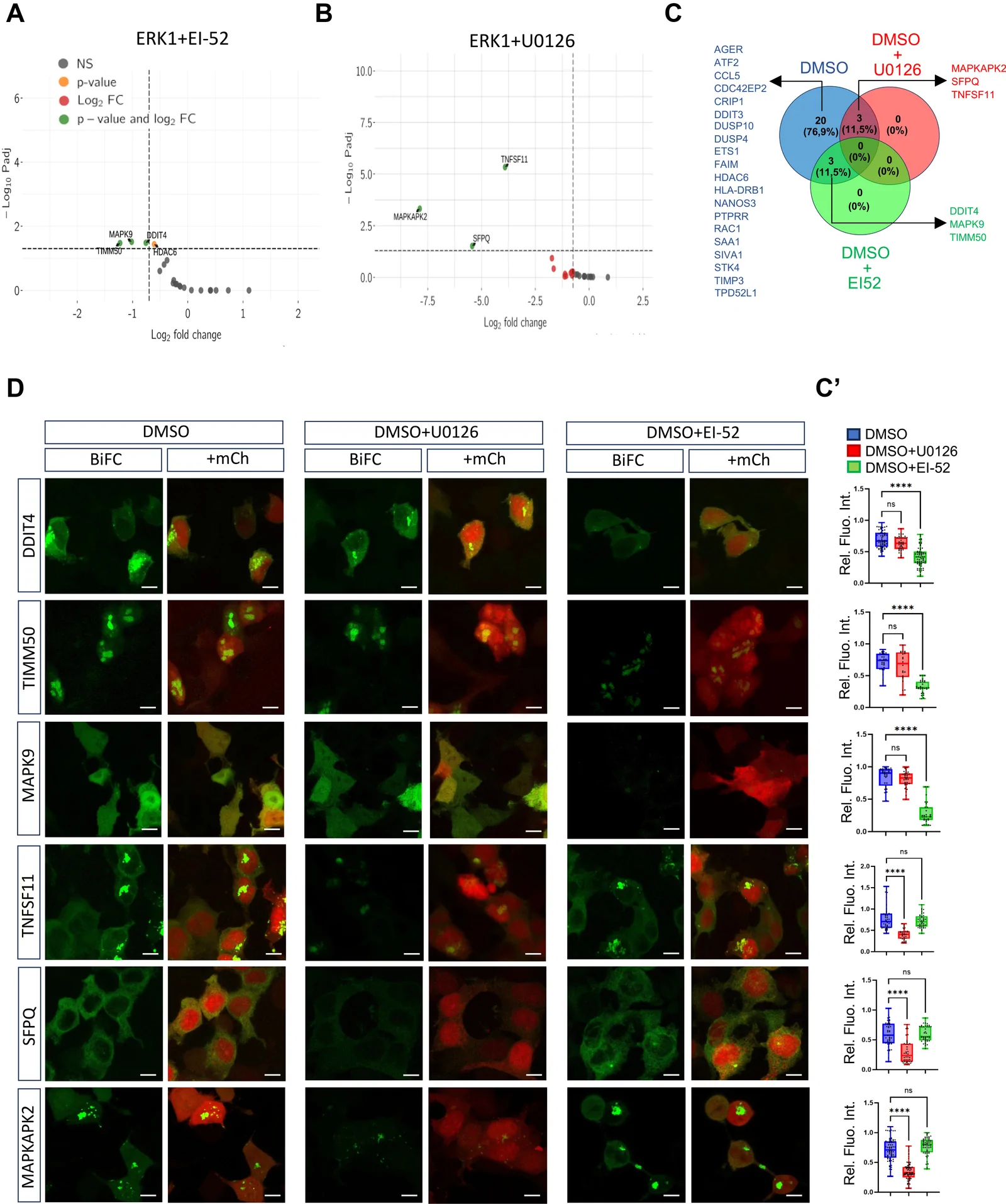
**Effects of the EI-52 and U0126 molecules on the ERK1 interactome.***A*, Volcano plot of EI-52–affected interactions. *B*, Volcano plot of U0126-affected interactions. Significantly affected ERK1-positive interactions that met the log2FC (positive or negative but equal or superior to 0,70) and adjusted *p* value (equal or inferior to 0.05) criteria are highlighted in *green* and labeled. Interactions that met only the adjusted *p* value or Log2FC criterion are highlighted in *orange* or *red*, respectively. *C*, Venn diagram showing candidates that are specifically affected by EI-52 or U0126 out of the 26 ERK1 interactors identified by Cell-PCA. *D*, illustrative confocal acquisitions of individual BiFC assays between ERK1 and the selected drug-sensitive interactions. Constructs were cotransfected with the mCherry reporter for normalizing BiFC fluorescence between the three different conditions (DMSO, DMSO + EI-52, and DMSO + U0126). Drugs were added before transfection. The scale bar represents 10 μm. *Cʹ*, statistical quantification of BiFC signals (*y*-: relative fluorescence intensity) in the different conditions, as indicated. Significance was determined relative to the mean BiFC value calculated with the control DMSO condition and was evaluated using one-way ANOVA (number of biological replicates n ≥ 3; ∗∗∗∗*p <* 00.001; ns, nonsignificant). See also Figs. S7 and S8 and Tables S5–S10. BiFC, bimolecular fluorescence complementation; DMSO, dimethyl sulfoxide; ERK1, extracellular signal–regulated kinase 1; FC, fold change; PCA, protein complementation assay.

Finally, before validating the effect of EI-52 on individual interactions in live condition (see below), we tested whether the three EI-52–sensitive binding partners of ERK-1 (DDIT4, MAPK9, and TIMM50) could also be revealed by an alternative method. To this end, we performed coimmunoprecipitation (co-IP), using the interaction between ERK-1 and MyD88 as a positive control. Co-IP confirmed the positive interaction status of the three novel binding partners of ERK-1, establishing them as solid candidates to evaluate the effect of EI-52 (Fig. S8). The effect of EI-52 was tested by BiFC in live HEK293T cells, applying the same experimental conditions as for the Cell-PCA screen (see also the Experimental procedures section). As a specificity control, we crosstested U0126 and EI-52 against each other’s sensitive interactions. The specific effect of EI-52 on ERK1–MyD88, ERK1–DDIT4, ERK1–MAPK9, and ERK1–TIMM50 was reproduced in each individual BiFC assay, validating the conclusion raised from the Cell-PCA screen with the BiFC–EI-52–ERKeome library (Fig. 4, *C* and *C′*).

Overall, our results confirmed that Cell-PCA effectively revealed specific effects of EI-52 compared with U0126 within a candidate ERK1-sensitive interactome.

### Using the splitFAST2 system to analyze the inhibitory role of EI52 after protein complex formation

Most of the current assays for validating the role of a candidate drug rely on treatments administered before protein complex formation. These assays provide information on the capacity of the drug to inhibit the initiation of the interaction but not on its capacity to destabilize the protein complex once it is formed, which is an important parameter when considering the future therapeutic potential of a candidate molecule. This constraint applies particularly in the case of Cell-PCA, since BiFC is stabilizing the protein complex with the formation of irreversible interactions between the two nonfluorescent fragments. Therefore, BiFC is not appropriate for evaluating the capacity of a molecule to destabilize a dimeric protein complex after its formation.

To tackle this issue, we decided to introduce a reversible complementation system called splitFAST2. The FAST (Fluorescence-Activating and absorption-Shifting Tag) protein can lead to instantaneous fluorescence upon noncovalent binding of fluorogenic 4-hydroxybenzylidene rhodamine (HBR) derivatives that are inherently nonfluorescent (18). Several FAST variants have been engineered to use different chemogenetic reporters (19). In addition, FAST can also be reconstituted upon the complementation of two subfragments, allowing analyzing PPIs in live cells (20, 21). The so-called splitFAST2 enzyme has recently been modified to display higher brightness and lower self-complementation (22).

The proof of concept and experimental parameters for using splitFAST2 were first established with the ERK1–MyD88 complex, with or without EI-52 and U0126 molecules. Here, the goal was to test the ability of EI-52 to destabilize its target protein complex postformation. Practically, BiFC and splitFAST2 constructs were cotransfected in HEK293T cells and cultured for 12 h without any drug, then incubated for an additional 8 h with the drug (or dimethyl sulfoxide [DMSO] as a control) before assessing the effect on each protein complex. The HBR ligand was added just before live-cell imaging at the confocal microscope to reveal preformed complexes (see the Experimental procedures section). Control experiments with DMSO or DMSO and U0126 showed overlapping BiFC (green) and splitFAST2 (red) signals, confirming that interaction properties between ERK1 and MyD88 remained unchanged under these conditions (Fig. 5, *A*–*C* and *F* and Movies S1–S6).

**Figure 5:**
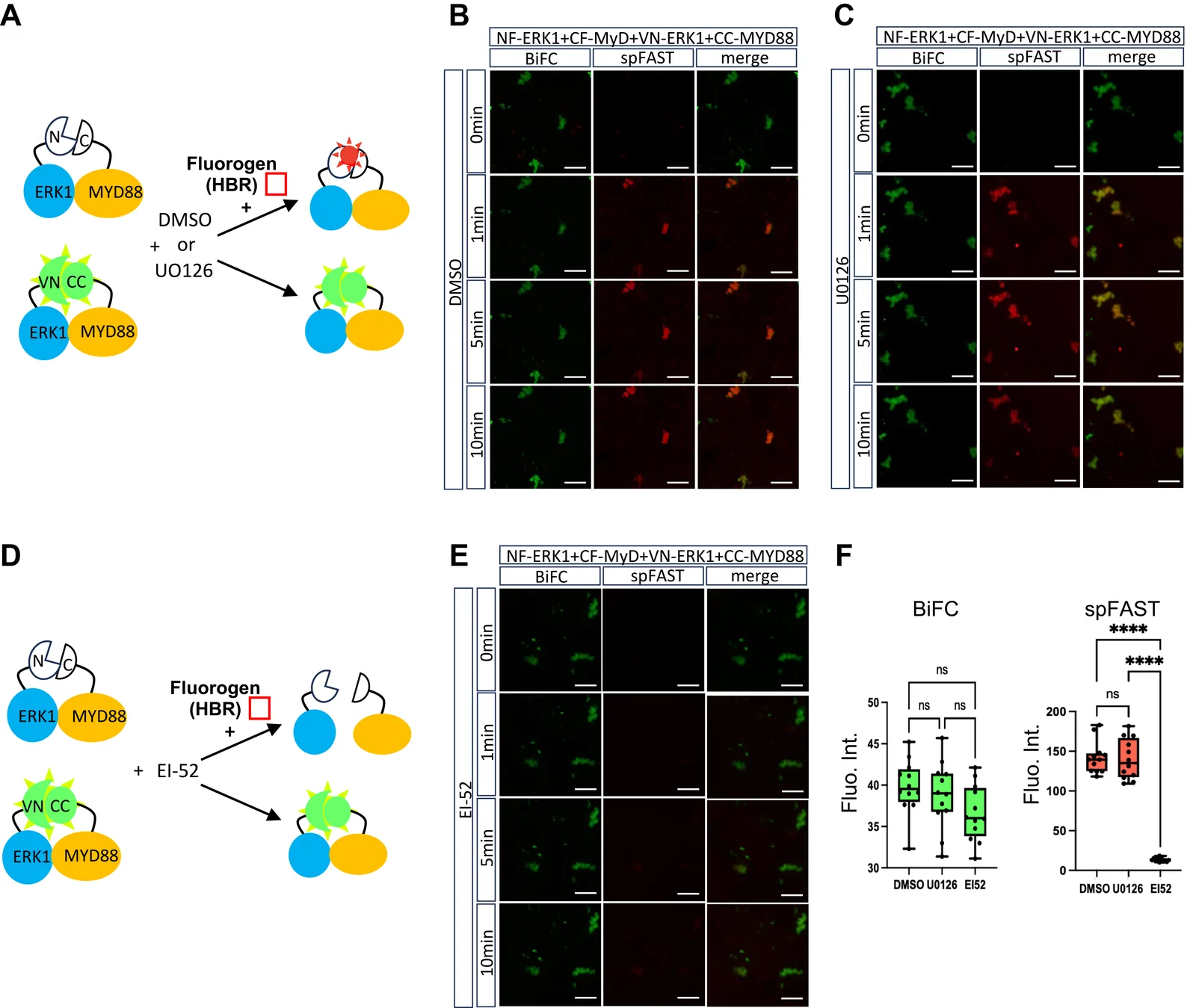
**Establishing splitFAST2 with the ERK–MYD88 complex.***A*, scheme summarizing the neutral effect of DMSO and U0126 on the ERK1–MYD88 interaction when assessed by doing BiFC and splitFAST2, and when tested after complex formation. *B* and *C*, illustrative confocal acquisitions of live HEK293T cells transfected with the splitFAST2 (NFAST-ERK1 and CFAST-MYD88, *red*) and BiFC (VN-ERK1 and CC-MYD88, *green*) constructs before adding DMSO (*B*) or DMSO with U0126 (*C*), as indicated (see also the Experimental procedures section). Acquisitions are shown at 0, 1, 5, and 10 min after the addition of the *red* HBR ligand. Neither BiFC nor splitFAST2 signals were affected in any condition (n = 3). *D*, scheme summarizing the potential effect of EI-52 on the ERK1–MYD88 interaction when assessed by doing BiFC and splitFAST2 and when tested after complex formation. The reversible nature of splitFAST2 allows assessing the potential destabilizing effect of EI-52 postcomplex formation. In contrast, the irreversible nature of BiFC does not allow assessing this level of inhibition. *E*, illustrative confocal acquisitions of live HEK293T cells transfected with the splitFAST2 (NFAST-ERK1 and CFAST-MYD88, *red*) and BiFC (VN-ERK1 and CC-MYD88, *green*) constructs before adding EI-52, as indicated (see also the Experimental procedures section). Acquisitions are shown at 0, 1, 5, and 10 min after the addition of the *red* HBR ligand. splitFAST2 signals were specifically affected upon the addition of EI-52 (n = 3). *F*, statistical quantifications of BiFC and spFAST in the different conditions, as indicated (∗∗∗∗*p <* 00.001; ns, nonsignificant). The scale bar represents 10 μm. See also Movies S1–S3. BiFC, bimolecular fluorescence complementation; DMSO, dimethyl sulfoxide; ERK1, extracellular signal–regulated kinase ; HBR, hydroxybenzylidene rhodamine; HEK293T, human embryonic kidney 293T cell line.

In contrast, incubating preassembled ERK1–Myd88 complexes with DMSO and EI-52 led to a strong decrease in splitFAST2 signals, whereas BiFC remained unchanged (Fig. 5, *D*–*F* and Movies S7–S9). Thus, these results demonstrate that EI-52 can destabilize the established ERK1–Myd88 complexes. Importantly, they also confirmed that splitFAST2 was appropriate for revealing such inhibitory effects.

Next, we combined BiFC and splitFAST2 to analyze the effect of EI-52 in the context of the EI-52–sensitive interactions found with Cell-PCA (ERK1–DDIT4, ERK1–TIMM50, and ERK1–MAPK9). As a control, we also tested the effect of EI-52 on one novel U0126-sensitive interaction (ERK1–SFPQ). As expected, BiFC was never affected in any cases (Fig. 6, *A*–*D*). In contrast, splitFAST2 signals were systematically affected in the context of EI-52–sensitive interactions (Fig. 6, *A*–*C*). These effects appeared specific since splitFAST2 signals resulting from ERK1–SFPQ remained unchanged (Fig. 6*D*).

**Figure 6:**
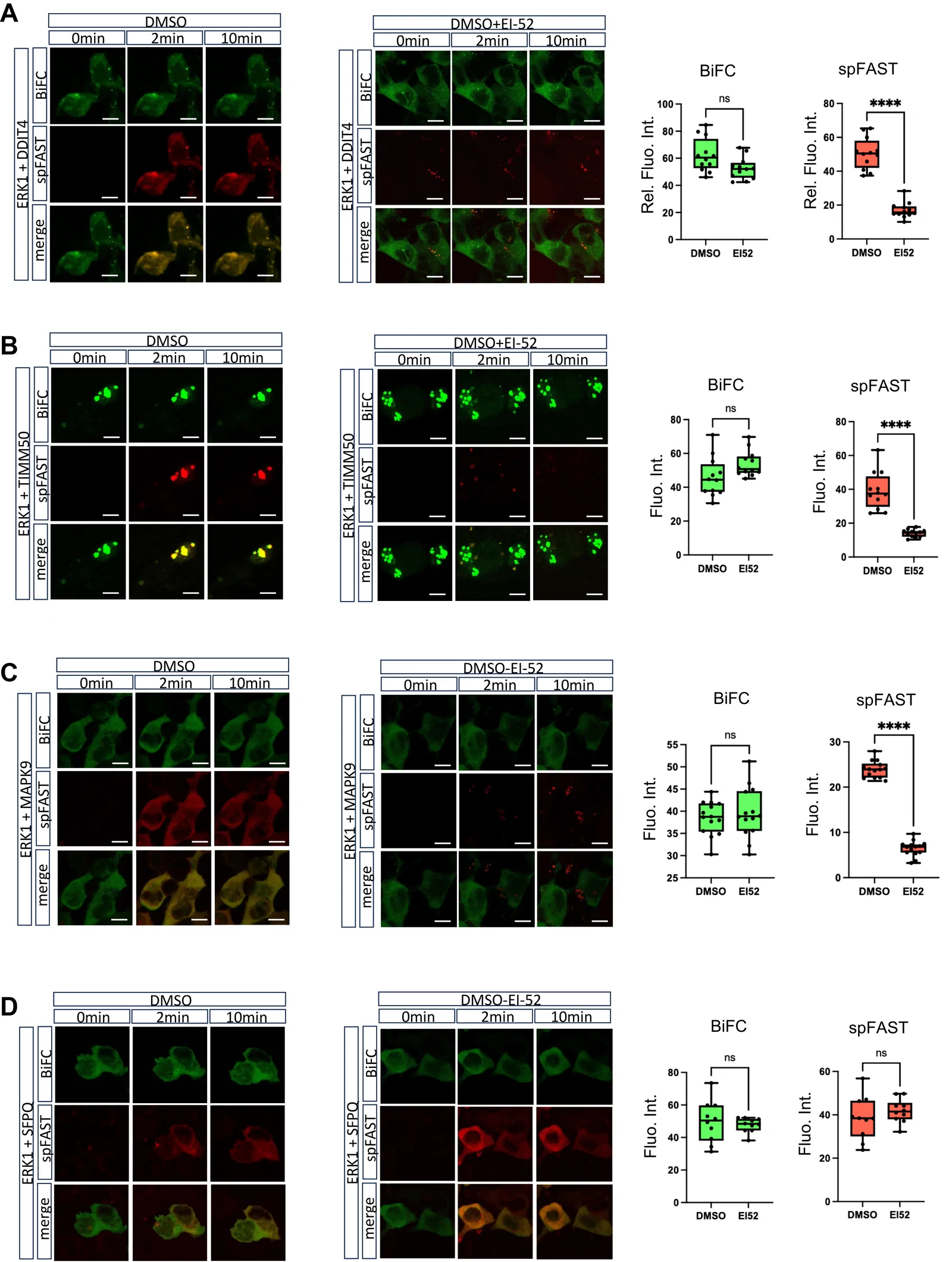
**Testing the effect of EI-52 on the novel drug-sensitive interactions of ERK1 postcomplex formation.***A*, effect of DMSO or DMSO + EI-52 on the ERK1–DDIT4 interaction. *B*, effect of DMSO or DMSO + EI-52 on the ERK1–TIMM50 interaction. *C*, effect of DMSO or DMSO + EI-52 on the ERK1–MAPK9 interaction. *D*, effect of DMSO or DMSO + EI-52 on the ERK1–SFPQ interaction. Cells were cotransfected with BiFC and splitFAST2 constructs, and drugs were added as in Figure 5. Illustrative confocal acquisitions are provided at different time points after the addition of HBR, as indicated (n ≥ 2). The scale bar represents 10 μm. Statistical quantifications of BiFC and splitFAST2 signals are shown on the *right* for each condition. BiFC, bimolecular fluorescence complementation; DMSO, dimethyl sulfoxide; ERK1, extracellular signal–regulated kinase 1; HBR, hydroxybenzylidene rhodamine.

Overall, results obtained with splitFAST2 revealed that EI-52 had the capacity to destabilize each tested sensitive interaction postcomplex formation. They also demonstrate complementarity with BiFC to better understand the range of activity of inhibitory molecules.

## Discussion

### A novel Cell-PCA design to capture drug-sensitive partners of ERK1 in human living cells

In this study, we presented an updated version of Cell-PCA (15) that relies on novel tools to make the approach more robust and specific. Alternative methods have been developed for interactome drug screening in living cells, including mammalian two-hybrid (M2H) and proximity labeling (BioID/APEX). Compared with Cell-PCA, M2H does not reproduce the normal subcellular environment for the readout of all PPIs and is most often used for testing candidate molecules on preselected pairs of interacting proteins (23). Proximity labeling provides valuable insights into drug sensitivity within endogenous interactomes (*e.g*., (24, 25); however, it necessitates access to high-performance mass spectrometers and large amounts of sample material for quantitative proteomics. Moreover, the changes detected by proximity labeling may stem from alterations in endogenous protein expression rather than true variations in PPI properties. Cell-PCA thus represents a good compromise, enabling rapid, sensitive (because of the stabilization of transient and weak PPIs by the BiFC), and large-scale screening of candidate molecules across hundreds to thousands of specific PPI pairs in living cells.

As a proof of concept, we established a BiFC HEK293T cell population for screening new interactions of the ERK1 regulator in the context of drug-sensitive functions. To this end, the ERKeome was chosen to cover MAPK signaling pathway function and, importantly, to be enriched in physiological responses induced by the EI-52 molecule. The new screening strategy was based on a systematic comparison with the VN fragment alone and on several additional improvements, including a novel inducible expression system, a novel sequencing protocol, and a novel statistical pipeline to select interactors with high confidence. The screen was performed in an easy-to-transfect cell line. Although it remains to be firmly established, Cell-PCA could in theory also be performed in cells that are not compatible with transfection (*i.e*., primary cells), thanks to the high sensitivity of BiFC. In this context, the VN-bait fusion construct could be delivered by lentiviruses (with high multiplicity of infection to get several copies in transduced cells) and the constructs induced with higher doses of dox.

This work led to the capture of 26 interactions out of the 907 ORFs present in the BiFC–EI-52–ERKeome cell population in the absence of U0126 and EI-52. This moderate rate of positive interactions could be explained by several reasons. First, it reflects the specificity of the approach. Second, less than 11% of the 907 integrated ORFs have already been captured as ERK1 interactors by other approaches (https://thebiogrid.org/111581/summary/homo-sapiens/mapk3.html), highlighting that the drug-sensitive ERKeome did not cover many known ERK1 interactions. Third, the BiFC screen was performed under basal and not strongly induced MAPK signaling conditions (with no growth factor supplementation in the cell culture medium), as attested by the equal distribution of VN–ERK1 in the nucleus and cytoplasm of transfected HEK293T cells. These experimental conditions could explain that the EI-52–sensitive control MyD88 (that is interacting with activated phospho-ERK1 only) was not captured in the Cell-PCA screen. Furthermore, most CC-ORFs are present as single-copy genomic insertions (see the Experimental procedures section). Because these insertion sites are random, expression levels can vary depending on the genomic locus. This limitation is avoided in individual BiFC assays using transient cotransfection of VN- and CC-fusion constructs, which generate significantly higher levels of BiFC complexes than the Cell-PCA approach. Nevertheless, the most enriched GO term among the 26 captured ERK1 interactions was the “MAPK signaling pathway,” even though this term was not the most enriched in the baseline BiFC–EI-52–ERKeome library.

Among the 26 ERK1-positive ORFs, half of them were described as direct ERK1 interactors and/or as functionally interacting with the MAPK pathway in normal or oncogenic processes. In addition, the majority of positive ORFs (18/26) have a predicted ERK D-binding domain. Thus, our approach was successful in revealing novel ORFs as putative interaction partners of ERK1. These ORFs encode for proteins present in different cellular compartments, including the nucleus (such as AGER, ATF4, DUSP4, ETS1, HDAC6, or RAC1), the perinuclear region (PTPRR), the mitochondria (DDIT4 or TIMM50), or the cytoplasm (DDIT4, HDAC6, or SIVA1). These various profiles were also revealed in our individual BiFC assays with ERK-positive ORFs. Altogether, these observations underline that our cell expression system was sensitive enough to reveal diverse molecular and cellular functions of ERK1.

### Specificity of U0126 and EI52 inhibitory molecules

A key aspect in pharmacology is the molecular range of activity of cancer-inhibitory molecules for future therapeutic perspectives. In this study, we directly tackled this issue by constructing an ERKeome covering diverse cellular events induced upon treatment with either U0126 or EI-52. Surprisingly, very few interactions were affected by the drugs, and these effects were drug specific. Among the interactions affected by U0126, only MAPKAPK2 was previously described as forming a complex with ERK1 (26, 27). SFPQ was recently proposed to regulate ERK activity during oocyte maturation (28) and TNFSF11 (also named RANK) was described to be activated by ERK during osteoclast differentiation (29). In contrast to these interactions with no established role in oncogenesis, the novel EI-52–sensitive interactions captured in this work are associated with cancer progression. DDIT4 has recently been shown to stimulate epithelial–mesenchymal transition in lung adenocarcinoma through the MAPK signaling pathway (30), TIMM50 has been described to promote tumor progression *via* MAPK signaling in non–small cell lung cancers (31), and MAPK9 is the subject of targeting approaches for cancer therapeutic perspectives (see, *e.g*. (32)). Furthermore, EI-52 induced ectopic interactions with ERK1, whereas U0126 showed no such effect. The lack of ectopic interactions with U0126 suggests that unphosphorylated ERK1 might be less effective at engaging in PPIs. Overall, our findings indicate that our experimental approach was appropriate for tackling the issue of the molecular range of action of two different drugs in the case of ERK1, and we propose that it could apply for any other bait protein that is the subject of pharmacological targeting development.

### Toward complementary complementation approaches for revealing inhibitory effects pre- and postcomplex formation

A molecule with therapeutic potential is usually selected for its capacity to inhibit the formation of a specific PPI. Whether it could also destabilize the protein complex once it is formed is rarely considered. Still, this aspect is of critical importance when considering that (1) aberrant PPIs are, by definition, of a stable nature and (2) drugs are delivered postcomplex formation *in vivo*. Therefore, testing this potential by using a reversible complementation system is necessary for selecting the best potent molecules for future therapeutic perspectives. A unique reversible complementation system based on the split Nanoluciferase enzyme has been described in the literature (33), but this system has never been used for assessing complex disassembly upon drug treatment to our knowledge.

Here, we demonstrated that the splitFAST2 system was appropriate for revealing the destabilizing effect of EI-52 on four different interactions (ERK1–MyD88, ERK1–DDIT4, ERK1–TIMM50, and ERK–MAPK9). The absence of effect on the U0126-sensitive interaction (ERK–SFPQ) further illustrates the specificity of these effects. The finding that EI-52 could destabilize the ERK1–MYD88 interaction postcomplex formation is in accordance with its previously described inhibitory role against MyD88-induced cancers (12). It also illustrates that the affinity of EI-52 is strong enough to displace previously established interactions with the D recruitment site of ERK. Moreover, the effect on ERK–DDIT4, ERK–TIMM50, and ERK–MAPK9 complexes suggests that the antitumor activity of EI-52 could also potentially rely on its blocking activity toward these three additional cancer-promoting interactions of ERK. This remains to be firmly demonstrated in a cancer cell context.

In conclusion, combining our cell-based complementation assay with splitFAST2 offers a straightforward strategy to characterize the inhibitory profile of candidate molecules within physiologically relevant interactomes, paving the way for therapeutic development.

## Experimental procedures

### Cell lines

HEK293T and HEK293T–CC–ORFs cells were maintained in Dulbecco’s modified Eagle’s medium (DMEM-GlutaMAX-I; Gibco by Life Technologies) supplemented with 10% (v/v) heat-inactivated fetal bovine serum (Gibco), and 1% (v/v) penicillin–streptomycin (5000 U penicillin and 5 mg streptomycin/ml) at 37 °C with 5% CO_2_. LentiX-293T cells were cultivated in Dulbecco’s modified Eagle’s medium (DMEM-GlutaMAX-I, Gibco by Life Technologies) supplemented with 10% fetal bovine serum at 37 °C with 5% CO_2_.

### Plasmids and GFP antibody

A total of 984 ORFs from the V9.1 version of the hORFeome were fused at the 5′ end to the C-terminal part of the mCerulean gene (encoding the last 155-238 amino acids) using Gateway technology. For individual BiFC assays, constructs were cloned into the plix _403 vector (a gift from David Root; Addgene plasmid #41395; http://n2t.net/addgene:41395, accessed on July 13, 2020; Research Resource Identifier: Addgene_41395) and fused to the 5' end with either the N-terminal part of the VN gene or the C-terminal part of the mCerulean gene. The VN–ERK and CC–ORF constructs were placed under the same tet-off dox–inducible promoter. All constructs were sequenced and verified before use.

Primary rabbit GFP (1/1000 dilution; Invitrogen, A11122) and anti-rabbit-Alexa Fluor 488 were used to reveal VN–ERK1 and CC–ORF constructs.

### Establishment of the BiFC-ERKeome cell line

LentiX-293T cells were transfected with pLIX-CC-ORFs plasmids, the lentiviral packaging plasmid pCMVR8.74, and the lentiviral envelope plasmid pMD2.G using jetPRIME (Polyplus, Ref 114–15) and following the manufacturer's recommendations. After harvesting the lentiviral particles, they were transduced in HEK293T cells at a low multiplicity of infection (0.3) to approximate a one-gene-one-cell condition with ≥1000× representation. During transduction, the culture medium was supplemented with 4 μg/ml polybrene (TR-1003; Sigma–Aldrich) and replaced the next day. Two days later, selection of transduced cells was performed by adding 0.75 μg/ml of puromycin (Gibco, catalog no. A1113803) to the culture medium for one week, until the uninfected control cells completely died and the selected cells reached near confluency. The medium was changed daily during this selection process. The final amplified transduced cells were divided into aliquots of 1 × 10^6^ cells each and stored in liquid nitrogen for future screens.

### Cell-PCA screen

For each replicate of the screen, a new batch of frozen BiFC–EI-52–ERKeome cells (1 × 10^6^ cells/ml) was cultured three days before the beginning of the experiment. Subsequently, BiFC–EI-52–ERKeome cells were plated in 6-well plates (2.3 × 10^5^ cells per well). Two days later, at approximately 80% cell confluence, the cells were transfected with either pLIX-VN-ERK1 (500 ng) or pLIX-VN-stop (500 ng) bait plasmids using 2 μl of jetPRIME buffer and 200 μl of jetPRIME reagent (Ref 114–15; Polyplus Transfection) per well. A mix containing jetPRIME buffer, jetPRIME reagent, plasmid DNA with DMSO (conditions VN–ERK1 and VN) or DMSO + 6 μM EI52 or DMSO + 10 μM U0126 (condition with VN–ERK1) was incubated for 20 min at room temperature before transfection. During transfection, the medium was replaced with complete medium supplemented with 100 ng/ml dox and the DNA mix. After 24 h, all transfected cells were pooled and resuspended in 200 μl PBS containing 2.5% serum volume fraction. A total of 50,000 BiFC-positive cells were subsequently sorted using a BD FACS Aria II cell sorter (performed at the AniRA-Cytometry core facility of the SFR Biosciences UAR3444/US8). After each screening, the sorted cells were collected, and genomic DNA was extracted using the PureLink Genomic DNA Mini Kit (catalog no. K182001; Invitrogen) following the manufacturer’s instructions. The extracted genomic DNA was then used for library preparation and was subjected to next-generation sequencing by in-house sequencing platform (PSI, IGFL).

### Nanopore long-read sequencing and identification of the positive hORFs

DNA extracts were quantified using a Qubit 4.0. Primers were designed on plix plasmid sequence in order to specifically amplify CCORFs inserted in the pLIX plasmid. The forward primer was designed in the CC domain (5′ GACGAGCTGTACAAGAGATC 3′) and the reverse primer in the hPGK promoter (5′ AACGGACGTGAAGAATGTG 3′) of the pLIX plasmid. CCORFs were amplified by long-range PCR in 26 cycles using LongAmp Hot Start Taq 2X Master Mix (New England Biolabs). For each sample, the totality of the DNA extracted was amplified in a multiple tubes PCR approach (1–4 PCR tubes according to the quantity of DNA available per sample). In a final volume of 30 μl, each PCR tube contained 50 ng of DNA, 15 μl of Long Amp MasterMix, and 1 μl of each forward and reverse primer at 10 μM. PCR conditions were as follows: initial denaturation step at 95 °C for 1 min, 26 cycles with denaturation step at 95 °C for 20 s, annealing step at 55 °C 30 s and elongation step at 65 °C for 4 min, and final elongation for 5 min at 65 °C. For each sample, all PCR tubes were pooled and purified using SPRI beads (Beckman Coulter) in ratio 1.7X. Final elution was performed in 30 μl using ultrapure water. Purified amplicons were quantified with Qubit 4.0 and quality assessed by TapeStation 4150 analyses (D5000 reagents and screentape; Agilent Technology). Oxford Nanopore Technologies (ONT)–barcoded libraries were constructed according to the ONT Native Barcoding Amplicon protocol with the SQK-LSK109 kit and EXP-NBD104. To initiate the library construction, 200 ng of amplified DNA per sample were used for End-Prep step. After native barcode ligation, maximum 24 quantified libraries (40 ng per sample) were pooled in an equimolar manner. The pooled library was finalized by ligation of sequencing adaptor before being deposited on a flowcell R9.4.1 (FLO-MIN106) for sequencing with Mk1C device. Fastq reads were generated by MinKNOW 21.10.8 using guppy version 5.0.17 for basecalling.

To analyze the data, ONT reads were filtered on their quality to retain the best quality reads (>Q12). A first BLAST approach against the pLIX plasmid was used to keep only those reads with the expected plasmid sequences at both ends. This step eliminates any artifactual reads resulting from PCR amplification of genomic fragments. A second BLAST approach was then performed using the hORFeome v8 reference database. Reads with a match on a CCORF in the database with at least 85% identity, and with an alignment length between 90% and 110% of the CCORF length, were assigned to the first BLAST hit (max_target_seq parameter set to 1). In this way, a read is assigned to only one CCORF. The BLAST parameters were chosen in order to take into account the amplification and ONT sequencing error rates. The BLAST results for all reads were then aggregated to generate a count table of CCORFs/genes detected per sample. In some cases, the parameters chosen for the BLAST approach are not able to differentiate between certain CCORFs. In order to identify these closest CCORFs, a BLAST of the hORFeome V8 DB against itself was performed using the same approach and parameters as for reads. All raw data are provided in Table S6.

### Identification of ERK1-interacting ORF candidates (DESeq2)

All analyses were performed with DESeq2 (v.1.46.0), a package for differential expression analysis from the R programming language (v.4.4.0), included as part of the Bioconductor project (http://www.bioconductor.org/packages/release/bioc/html/DESeq2.html and (34)). DESeq2 utilizes a custom class object called DESeqDataSet, which stores the data required to perform the differential expression analysis. Two input files are required: the first containing the un-normalized counts data and the second containing a table with sampleinformation.

Clustering analysis was conducted to assess similarities between samples. A heatmap of the sample-to-sample distance matrix was created using the pheatmap and RColorBrewer packages. The darker the color, the closer the samples are to each other. The distance matrix was computed on the transformed counts data using the variance-stabilizing transformation approach, which aims to remove the dependence of the variance on the mean.

Complementary to the clustering analysis, a principal component analysis (35) was applied to the transformed counts data to visually inspect the presence of batch effects. Principal component analysis is a dimension reduction technique that projects a data set onto a subspace of lower dimension described by an orthogonal basis. The plotPCA function of the DESeq2 package can be used to project the variance-stabilized transformation data onto the two-dimensional plane formed by the first two principal components, namely principal component 1 and principal component 2. The percentage on each axis label represents the fraction of total variance explained by each principal component.

After obtaining the list of candidates from the DESeq2 test for differential expression, two filtering criteria were further applied to discriminate ORFs: a log2 FC greater than 0.70 (log2FC ≥ 0.70) and an adjusted *p*-value smaller than 0.05 (*P*-adjusted ≤ 0.05).

A volcano plot was used to visualize the differentially expressed ORF candidates. Here, a custom function (lscript VolcanoPlot.R), based on the EnhancedVolcano package, was used to adapt the graph. The vertical line marks the log2 FC cutoff. The vertical line marks the adjusted *p* value cutoff in logarithmic scale, that is −log10(0.05).

To reproduce the results of the DESeq2 analysis, an R script (main.R) is available on the following GitLab repository: https://gitbio.ens-lyon.fr/igfl/merabet/BIFCScreen. As an alternative, the repository also provides a link to a Docker image.

### Over-representation analysis

An ORA was applied to gauge whether the size of the set of ERK interactors detected with the « no drug » ERK screen is significant or not, given how many known ERK interactors are present in the ERKeome library.

The ORA method (1) proceeds as follows. Consider a set of n differentially expressed genes, among which k genes belong to a gene set of interest, and a reference list of N genes among which M genes are annotated to the same gene set of interest. The ORA method assesses if the gene set of interest is significantly over-represented (enriched) in the list of differentially expressed genes using a hypergeometric distribution. The corresponding *p* value is defined as follows: *p* = 1 – P(X < k), where X is a random variable that follows the hypergeometric distribution H(N,M,n) and P is the probability mass function of X. With this definition, the *p* value answers the question of whether the gene set of interest is represented in the set of differentially expressed genes at a frequency greater than that would be expected by chance. When several gene sets are tested, the *p* values are further adjusted for multiple hypothesis testing.

Here, the reference list is the BiFC–EI-52–ERKeome library (N = 907), among which M = 95 are annotated as ERK interactors. The size of the set of differentially expressed genes, obtained from the BiFC screen, equals n = 26, among which k = 7 are ERK interactors. The ORA method implemented by the R package clusterProfiler (36) was used to produce the raw output (Table S5). The *p* value computed for the set of ERK interactors equals 0.0137, thus indicating a significant enrichment. This result should be interpreted in light of the fact that the ORA method is typically applied to a large number of gene sets of interest (GO terms), with the resulting *p* values then adjusted to account for multiple testing.

### Coimmunoprecipitation

HEK293T cells were seeded on 6-well plates at 5 × 10^5^ cells per well. One day later, a total of 2 μg of plasmid DNA (1 μg of pLIX-ERK-HA and 1 μg of pLIX-mCherry, pLIX-CC-DDIT4, pLIX-CC-MAPK9, pLIX-CC-MYD88, or pLIX-CC-TIMM50) was transfected per well using jetPRIME (Polyplus Transfection, 114-15). The cells were treated 3 h after transfection with 500 ng/ml of dox. Twenty-four hours after transfection, cells were washed and harvested in 1× PBS, and cell pellets were resuspended in Nonidet P-40 buffer (20 mM Tris, pH 7.5, 150 mM NaCl, 2 mM EDTA, and 1% Nonidet P-40) supplemented with protease inhibitor cocktail (Sigma–Aldrich, P8340). The protein extract (1 mg) was incubated with 25 μl of anti-HA magnetic beads (Thermo Fisher Scientific, 88836) for 4 h at 4 °C under gentle rotation. Beads were then washed three times with NP-40 buffer and directly resuspended in Laemmli sample buffer for immunoblotting analysis. For input evaluation, 25 μg of whole cell lysate was loaded onto the gel.

### Western blot

Protein samples (25 μg of input or 40 μl of IP eluate) were separated on 10% SDS-PAGE and transferred onto polyvinylidene fluoride membrane (Merck Millipore; IPVH00010). Membranes were blocked for 1 h at room temperature in Tris-buffered saline containing 0.1% Tween-20 and 1% bovine serum albumin. Membranes were then incubated overnight at 4 °C with primary antibodies, followed by 1 h at room temperature with horseradish peroxidase–conjugated secondary antibodies. Signal detection was performed using an enhanced chemiluminescence system (Merck Cytiva; RPN 2236). Primary antibodies used were HA (1/500, ABCD, and AF291), rabbit GFP (1/1000 dilution; Invitrogen, A11122), and H3 (1/20,000 dilution, ab1791). Horseradish peroxidase–conjugated secondary antibodies were anti-rabbit IgG (1/5000 dilution; Promega Jackson Immunoresearch, 711-035-152).

### Individual BiFC validation in live cells

#### Without inhibitor

HEK293T cells were seeded on glass coverslips in 6-well plates (3.5 × 10^5^ cells per well). One day later, a total of 500 ng of plasmid DNA (250 ng of pLIX–VN–ERK and 250 ng of pLIX–CC–ORF) was transfected per well using jetPRIME (reference: 114–15; Polyplus Transfection) and following the manufacturer’s instructions. The cells were simultaneously cotransfected with 100 ng of pLIX-mCherry plasmids to assess transfection efficiency and treated with dox (100 ng/ml final). After 18 h of incubation, the cell-coated coverslip was removed and carefully mounted on a glass slide using a 4% formaldehyde solution for imaging under a Zeiss LSM780 confocal microscope (Carl Zeiss).

#### With inhibitor

HEK293T cells were seeded (2.5 × 10^5^ cells) in a μDish IBIDI (reference: 81156). For U0126 treatment, it was crucial to pretreat the cells before transfection. One day after plating, cells were pretreated with U0126 (20 μM final) or DMSO (20 μM final) for control. The following day, a total of 600 ng of plasmid DNA (250 ng of pLIX–VN–ERK, 250 ng of pLIX–CC–ORF, and 100 ng of pLIX-mCherry) was transfected per well using jetPRIME (reference: 114–15; Polyplus Transfection) and following the manufacturer's instructions. During transfection, the cells were simultaneously treated with U0126 (30 μM final) or DMSO (30 μM final) and dox (100 ng/ml final). After 24 h of incubation, live cell imaging was performed using a Zeiss LSM780 confocal microscope (Carl Zeiss). For EI52, no pretreatment was required. The same conditions used for U0126 were applied without pretreating the cells. At the time of transfection, the cells were treated with EI52 (12 μM final) or DMSO (12 μM final) for control.

### splitFAST

HEK293T cells were seeded (2.5 × 10^5^ cells) in μDish IBIDI (reference: 81156) coated with poly-l-lysine the day before beginning the experiment. Once the cells reached approximately 80% confluence, they were transfected with splitFAST2 constructs (1 μg of pLIX-NFAST-ERK1 and 1 μg of pLIX-CFAST-ORF) using jetPRIME (reference: 114–15; Polyplus Transfection) according to the manufacturer's recommendations. As a control, cells were simultaneously transfected with BiFC constructs (200 ng of pLIX-VN-ERK1 and 200 ng of pLIX-CC-ORF) from the same partner pair. To study the dissociation of the splitFAST2 system, cells were treated with dox (500 ng/ml final) at the time of transfection. Twelve hours later, the medium was replaced with complete medium without phenol red to remove dox and phenol red (to avoid fluorescence interference with the HBR signal), and the cells were subsequently treated with EI52 (12 μM final) or U0126 (10 μM final) or DMSO (12 μM or 10 μM final) as controls. About 8 h after treatment, cells were imaged live under Zeiss LSM780 confocal microscope (Carl Zeiss). The optimal HBR fluorogen concentration required to visualize the association or dissociation of the splitFAST2 system was determined for each interaction tested. A 100 μl solution containing the appropriate concentration of fluorogen was added live just before doing confocal acquisitions.

### Fluorescence quantification analysis

Quantification was statistically analyzed using normality tests followed by a paired *t* test (two conditions) or an ordinary one-way ANOVA (three conditions) with Prism 10 (GraphPad).

A minimum of three biological replicates were applied for each experimental condition. Three to four confocal acquisitions were performed for each biological replicate. Pictures were acquired at the 40x objective, and each field contained approximately between 10 and 20 positive cells that were used for the fluorescence quantification. Quantification of BiFC was relative to transfection efficiency: a mean fluorescence intensity was determined for all mCherry-positive cells of the acquisition field, and this mCherry mean fluorescence was compared between the different conditions to re-evaluate the mean BiFC intensity when different. For example, if the “no-drug” condition had a mean mCherry value 1.5x weaker than the mean mCherry value of the “with-drug” condition, the mean BiFC value was readjusted accordingly (increased 1.5x in the “no-drug” condition). Such measurement of BiFC relative to transfection efficiency could not be performed in the double BiFC/splitFAST-transfected cells (but the specific variations of split-FAST signals confirmed that it was transfection independent). An average value deduced from the quantification of all positive cells was deduced for each acquisition field. These values were used for graph representation and statistical analyses.

### Clustering interactome and functional enrichment analysis

Functional networks of the ERK interactome were generated with STRING software (https://string-db.org/), based on an 0.15 interaction score of experimental evidence, database, and pathway co-occurrence. Network visualizations were built with Cytoscape free software conserving the default parameters and clustered according to their biological functional enrichment (https://cytoscape.org/). The GO-Term annotations and over-represented GO-Term related to biological process analysis were performed with Metascape (https://metascape.org/gp/index.html#/main/step1).

## Data availability

Data are available upon request.

## Supporting information

This article contains supporting information.

## Conflict of interest

The authors declare that they have no conflicts of interest with the contents of this article.
