# Supplementary material for: A sensitive interactome-based approach to characterize drug activity in living human cells
Source: J Biol Chem. 2026 Sep 17;302(11):113557. doi: 10.1016/j.jbc.2026.113557 (PMC13678014; doi:10.1016/j.jbc.2026.113557)
Supplement: Supplementary file 1 [file mmc10.pdf]

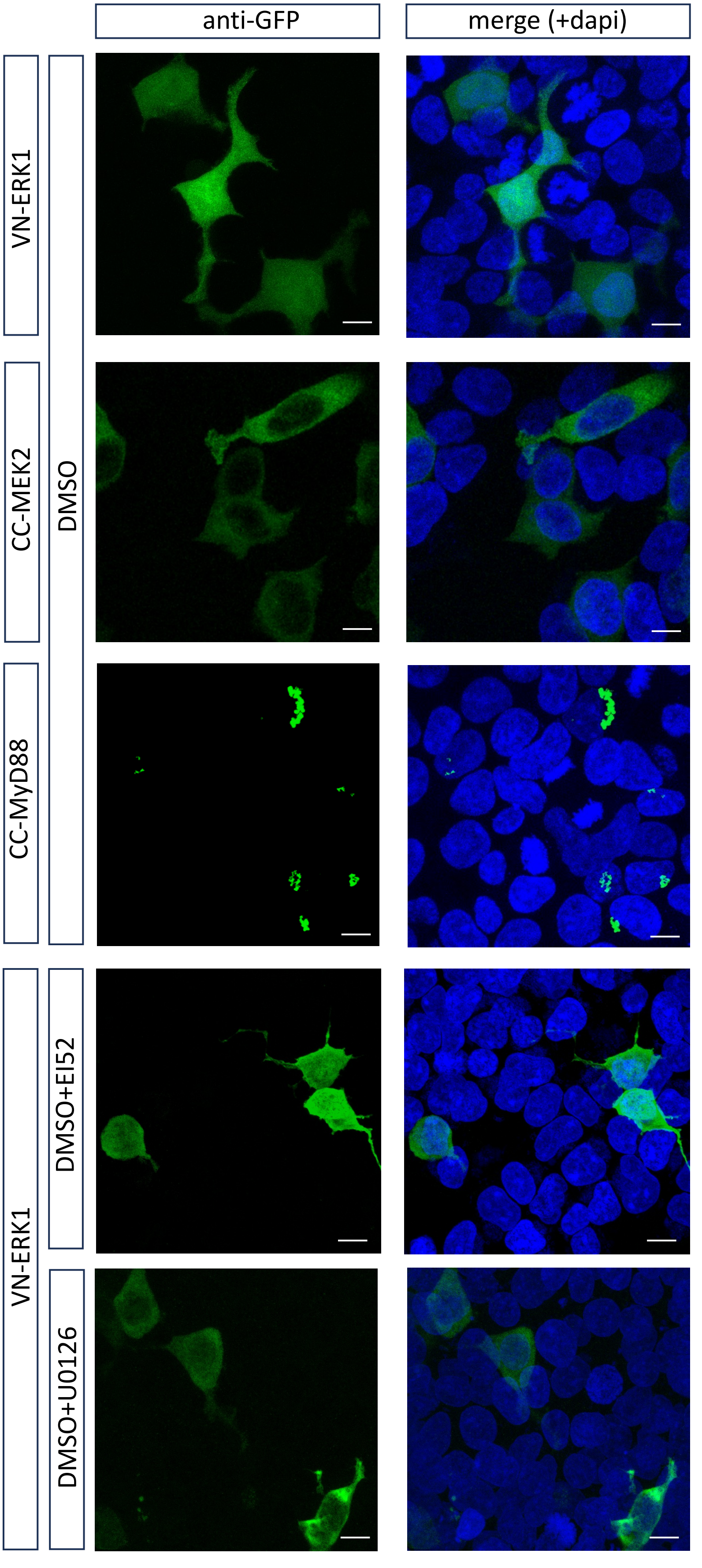

Supplementary Figure 1

Fluorescent immunostaining of the VN-ERK1, CC-MEK2 and CC-MyD88 constructs transfected in HEK293T cells with DMSO or DMSO and UO126 or EI52, as indicated. Constructs are revealed with an anti-GFP (green) recognizing the VN and CC fragments (rabbit anti-GFP from Invitrogen, A1122). Dapi (blue) stains for nuclei. Scale bar=10 μm.

A

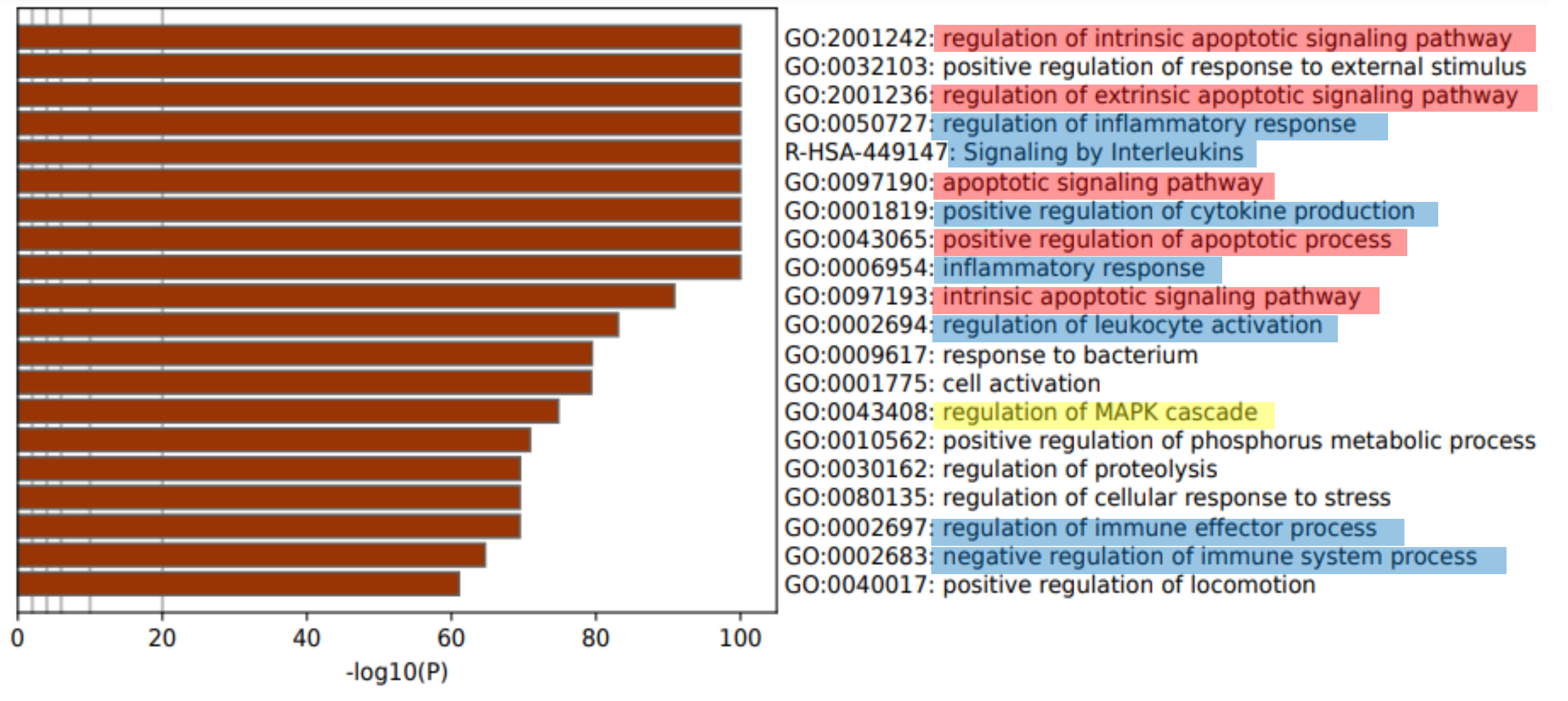

B

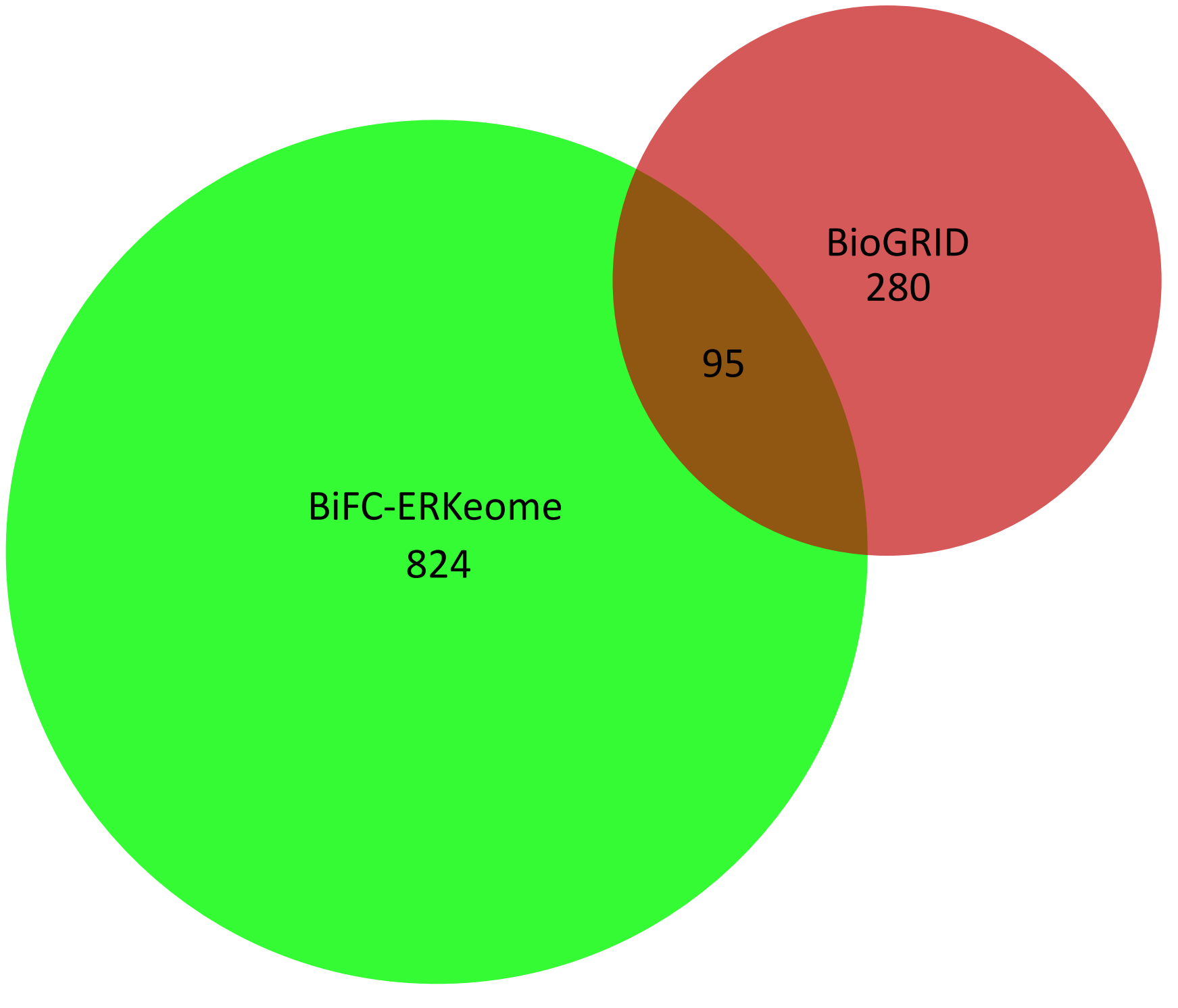

Supplementary Figure 2

A. GO term enrichment of the integrated BiFC-ERKeome in HEK293T cells. Color code indicates functionally related GO terms (apoptosis in red, MAPK pathway in yellow, immunity in blue). **B.** Venn diagram showing the overlap between the integrated BiFC-ERKeome and the known ERK1 interactome (as provided in the BioGrid database).

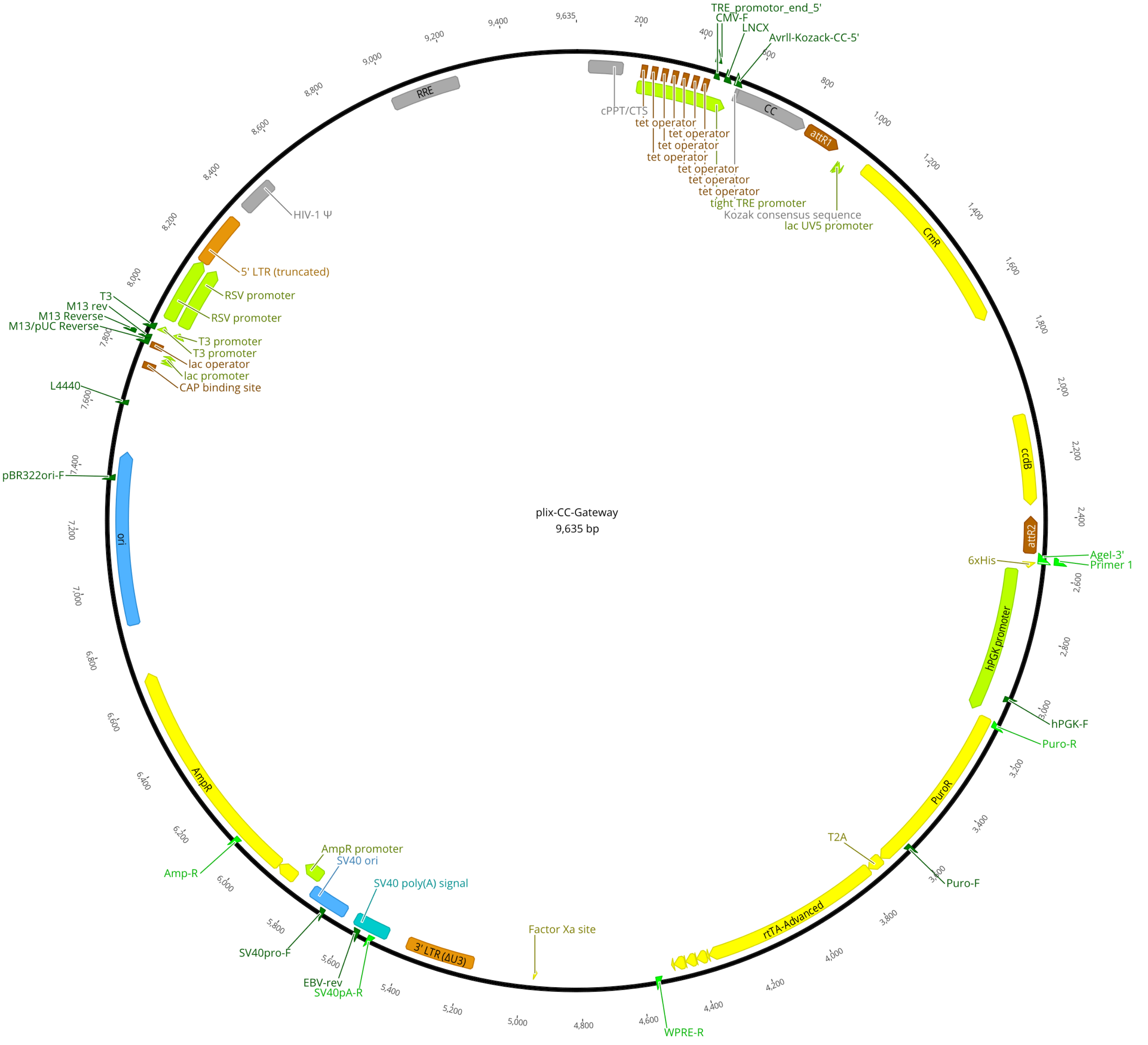

Supplementary Figure 3

Map of the pLix-CC-GW vector used to clone each candidate ORF in fusion with the C-terminal fragment of Cerulean (CC). GW: Gateway.

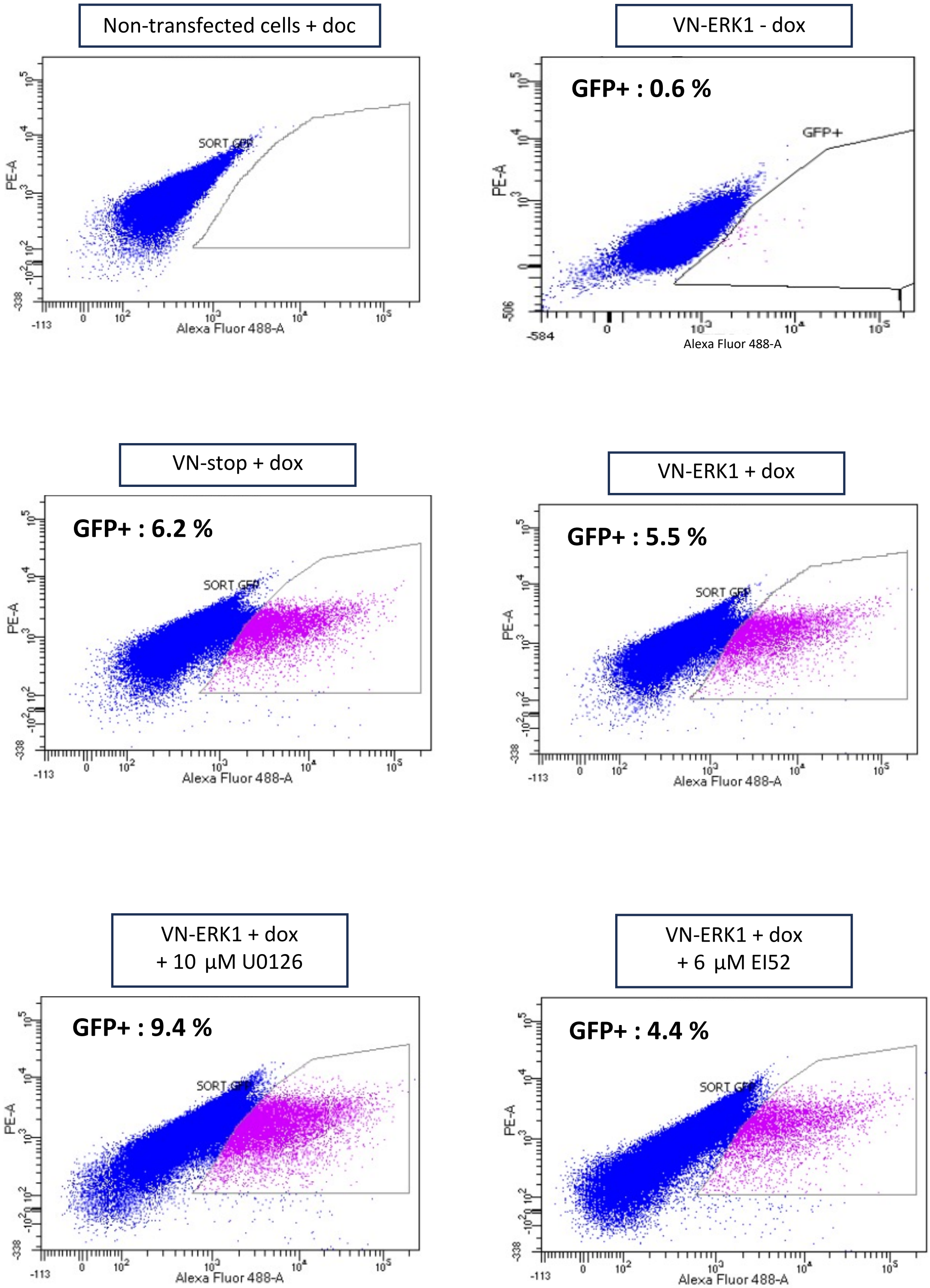

Supplementary Figure 4

Graphs showing the proportion of fluorescent cells when analyzed by the cell sorter in each condition, as indicated. The gate-filter used to specifically sort the fluorescent cells is indicated (black line).

A

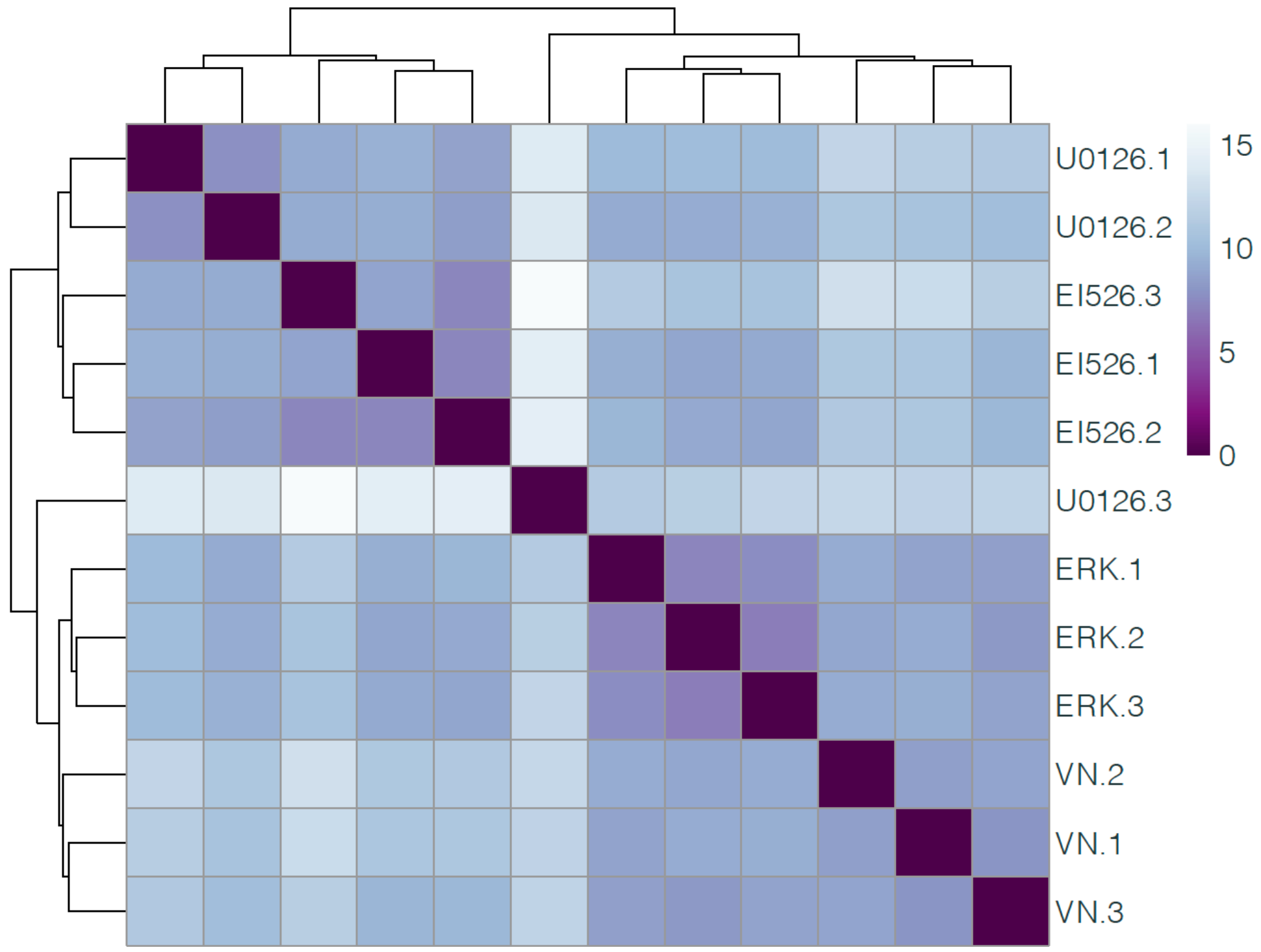

B

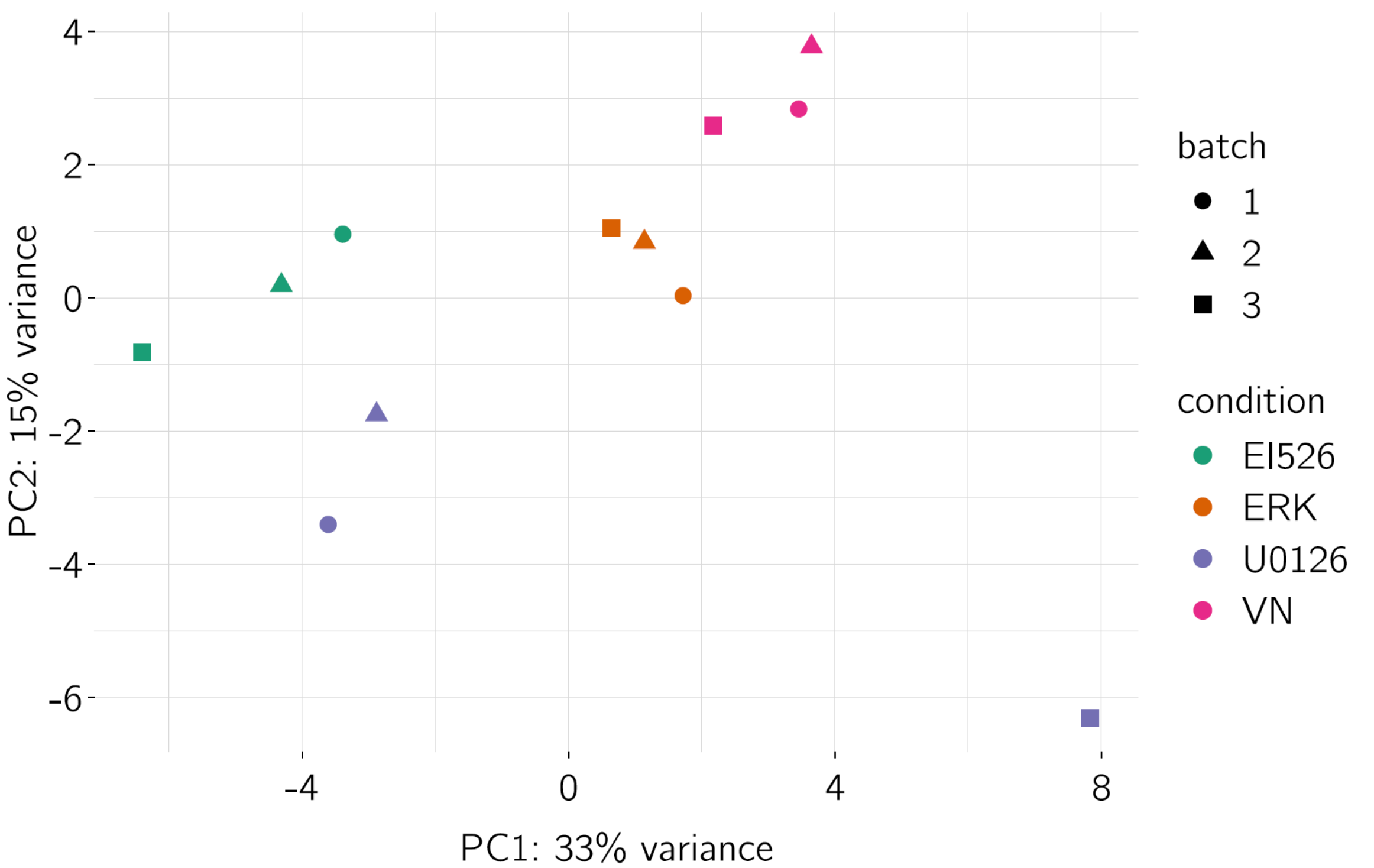

Supplementary Figure 5

Clustering of the different conditions. A. Hierarchical clustering of the different conditions and for the three biological replicates, as indicated. **B.** PCA of the different conditions (indicated by different colors) and for the three biological replicates (batch 1, 2 or 3).

ERK1-positive interactors		
Interactor	log2FoldChange	padj
AGER	4.52410212187222	0.0388496563548158
ATF2*	2.12931515685915	0.000251641041698506
CCL5	1.31977073281666	0.00147052230307002
CDC42EP2	1,88602015165334	11464776E-11
CRIP1	1.00179636184022	0.00334795364045253
DDIT3	2.13998231473065	0.00309815700553037
DDIT4	0.946122674610702	0.00054429313746805
DUSP10	0.865215780006314	0.043158122323803
DUSP4*	1.89205267657355	0.000251641041698506
ETS1*	1,10586044835991	4,24263103185244E-09
FAIM	0.817156224320465	0.0445599895174817
HDAC6*	0.765593516953451	0.016130786990156
HLA-DRB1	1,89511989730901	0,000048857671980901
MAPKAPK2*	5.69426464848748	0.000625682915115001
MAPK9	0,727881386689919	0,0260980995692692
NANOS3	1.02506602262822	0.000251641041698506
PTPRR*	3.91963602660258	0.000420401527073832
RAC1	1.21297325741334	0.000174354124671292
SAA1	3.01938242339636	0.0475847817846876
SFPQ	3.15769700542057	0.042432196070917
SIVA1	1.900487410846	0.000251641041698506
STK4	1,14216698057816	7,10182943321822E-06
TIMM50	1.53138243380952	0.00214487147502878
TIMP3	2.19163385621333	0.0475847817846876
TNFSF11	1.26919155128801	0.011157169543578
TPD52L1*	0,700042348179147	0,00570617653166801

Confidence score Scansite 4.0:

- high
- medium
- low
- No binding motif

Supplementary Figure 6

List of the 26 selected ERK1 interactors (with their corresponding log2 fold change and adjusted p-value from the BiFC screen with VN-ERK1 when compared to the VN fragment alone) analyzed with ScanSite for ERK D-binding domain(s). The confidence score is reflected by the color code. Interactors with an asterisk are known ERK1 cofactors described in Biogrid and/or String database.

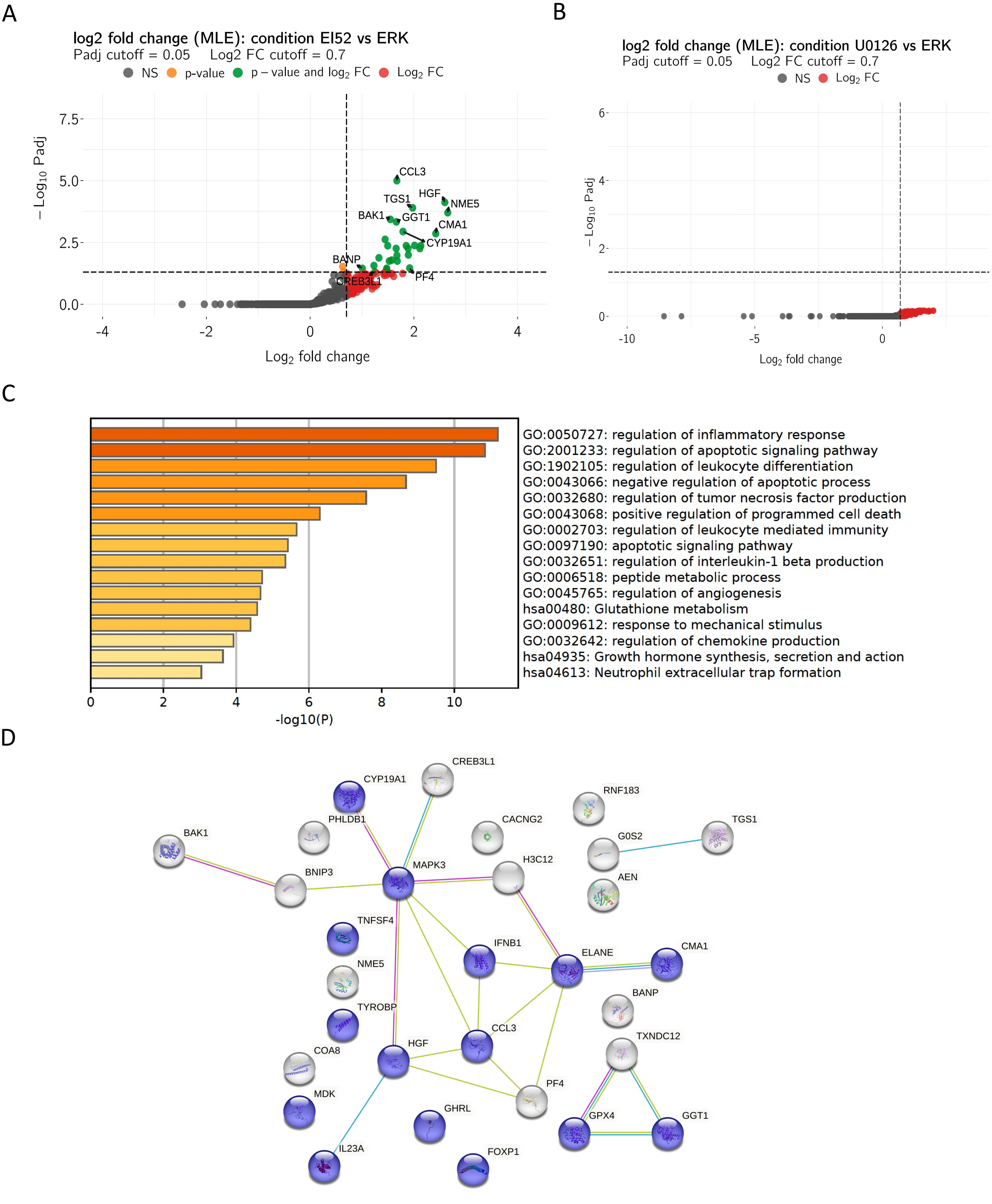

Supplementary Figure 7

ERK1 interactions induced in the presence of EI52 or U0126. A. Volcano plot of the 30 ERK1 interactions induced by EI-52. **B.** Volcano plot showing the absence of ERK1 interactions induced by U0126. **C.** Go-term analysis of the 30 EI-52-induced ERK1 interactions. **D.** Clustering of the 30 EI-52-induced ERK1 interactions. Proteins involve in inflammatory response (blue balls) form a major interaction network.

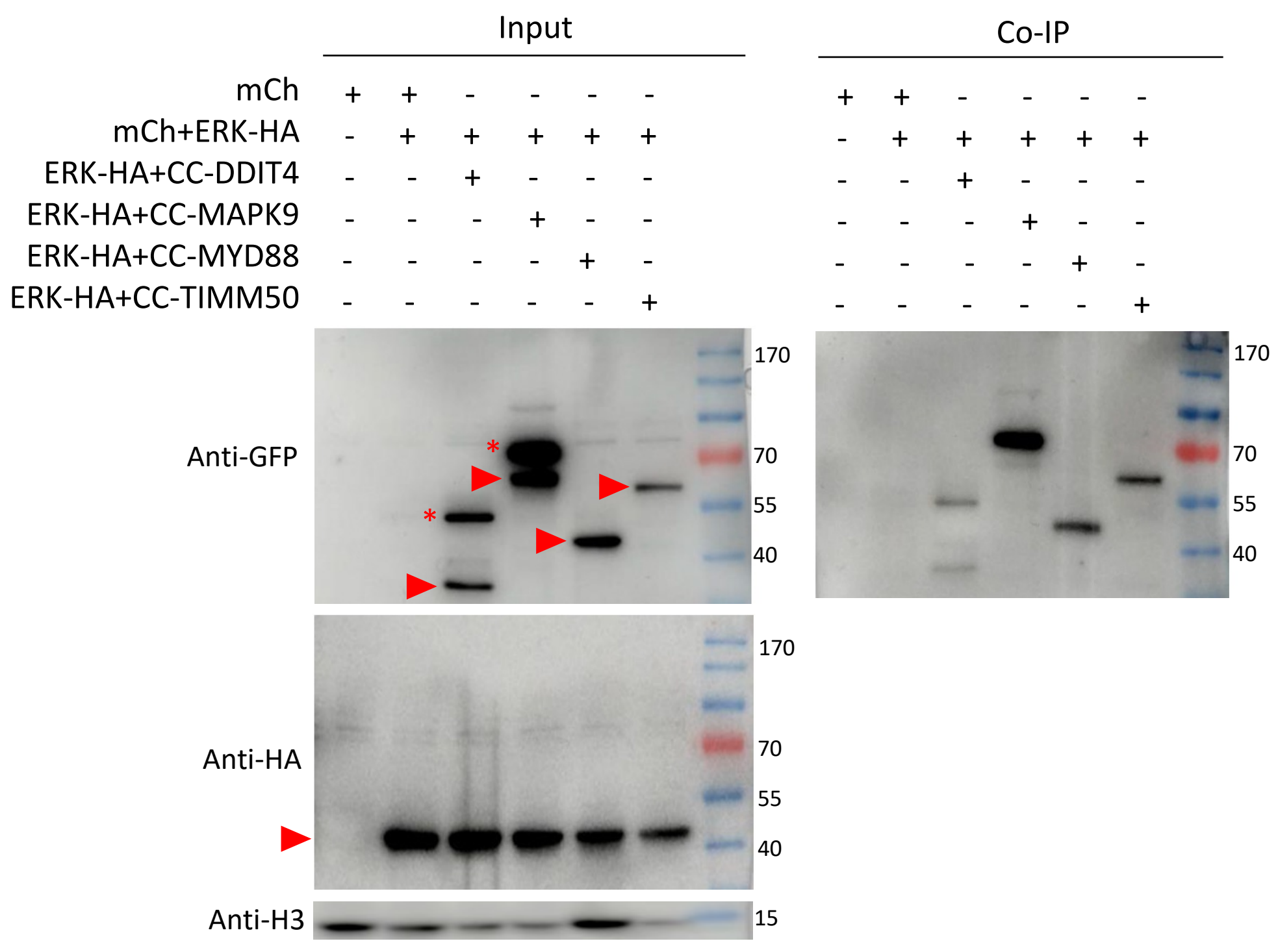

Supplementary Figure 8

Co-immunoprecipitation (co-IP) of HA-tagged ERK with candidate cofactors found to be EI-52 sensitive in the Cell-PCA screen. The co-IP is performed with an anti-HA antibody, and associated candidate cofactors are revealed with an anti-GFP recognizing the CC fragment (see also Materials and Methods). The different constructions expressed alone or in combination are indicated above the gels. Note the presence of two bands for CC-DDIT4 and CC-MAPK9. The expected size for CC-DDIT4 and CC-MAPK9 is 38kDa and 65kDa, respectively. The upper bands could correspond to forms with post-translational modifications such as sumoylation (but it remains to be demonstrated). Both forms of CC-DDIT4 interact similarly with ERK-HA, whereas CC-MAPK9 displays a stronger interaction with its higher molecular weight form. Expected (red arrowheads) and modified (red asterisks) sizes for the different constructs are indicated. mCh: control pLix vector expressing mCherry.
